# Proteomics-based identification of differentially abundant proteins reveals adaptation mechanisms of *Xanthomonas citri* subsp. *citri* during *Citrus sinensis* infection

**DOI:** 10.1186/s12866-017-1063-x

**Published:** 2017-07-11

**Authors:** Leandro M. Moreira, Márcia R. Soares, Agda P. Facincani, Cristiano B. Ferreira, Rafael M. Ferreira, Maria I. T. Ferro, Fábio C. Gozzo, Érica B. Felestrino, Renata A. B. Assis, Camila Carrião M. Garcia, João C. Setubal, Jesus A. Ferro, Julio C.F. de Oliveira

**Affiliations:** 1 Departamento de Ciências Biológicas (DECBI), Instituto de Ciências Exatas e Biológicas (ICEB), Universidade Federal de Ouro Preto (UFOP), Ouro Preto, MG Brazil; 2 Núcleo de Pesquisas em Ciências Biológicas (NUPEB), Universidade Federal de Ouro Preto, Ouro Preto, MG Brazil; 3 Departamento de Bioquímica (DBq), Instituto de Química (IQ), Universidade Federal do Rio de Janeiro (UFRJ), Rio de Janeiro, RJ Brazil; 4 Faculdade de Ciências Agrárias e Veterinárias de Jaboticabal, UNESP – Universidade Estadual Paulista, Departamento de Tecnologia, Jaboticabal, SP Brazil; 5 Instituto de Química, Universidade Estadual de Campinas (UNICAMP), Campinas, SP Brazil; 6 Departamento de Bioquímica (DB), Instituto de Química (IQ), Universidade de São Paulo (USP), São Paulo, SP Brazil; 7 Departamento de Ciências Biológicas (DCB), Universidade Federal de São Paulo (UNIFESP), Diadema, SP Brazil; 8 Biocomplexity Institute, Virginia Tech, Blacksburg, VA USA

**Keywords:** *Xanthomonas*, adaptation mechanisms, Biofilm, Iron uptake and metabolism, Plants’Oxidative burst, LPS modulation

## Abstract

**Background:**

*Xanthomonas citri* subsp. c*itri* (*Xac*) is the causal agent of citrus canker. A proteomic analysis under *in planta* infectious and non-infectious conditions was conducted in order to increase our knowledge about the adaptive process of *Xac* during infection.

**Results:**

For that, a 2D–based proteomic analysis of *Xac* at 1, 3 and 5 days after inoculation, in comparison to *Xac* growth in NB media was carried out and followed by MALDI-TOF-TOF identification of 124 unique differentially abundant proteins. Among them, 79 correspond to up-regulated proteins in at least one of the three stages of infection. Our results indicate an important role of proteins related to biofilm synthesis, lipopolysaccharides biosynthesis, and iron uptake and metabolism as possible modulators of plant innate immunity, and revealed an intricate network of proteins involved in reactive oxygen species adaptation during Plants` Oxidative Burst response. We also identified proteins previously unknown to be involved in *Xac*-*Citrus* interaction, including the hypothetical protein XAC3981. A mutant strain for this gene has proved to be non-pathogenic in respect to classical symptoms of citrus canker induced in compatible plants.

**Conclusions:**

This is the first time that a protein repertoire is shown to be active and working in an integrated manner during the infection process in a compatible host, pointing to an elaborate mechanism for adaptation of *Xac* once inside the plant.

**Electronic supplementary material:**

The online version of this article (doi:10.1186/s12866-017-1063-x) contains supplementary material, which is available to authorized users.

## Background

*Xanthomonas citri* subsp. *citri* (*Xac*) strain 306 pathotype A is the most aggressive causal agent of citrus canker, a disease that affects most citrus cultivars worldwide, causing considerable loss of fruits and their derivatives [1, 2]. After *Xac* invades plant tissue, localized induced canker symptoms correspond to watersoaking (3 Days After Induction - 3DAI), hyperplasia (5DAI), and necrosis (about 14DAI) [3]. The infection process culminates into rupture of the plant tissue and dispersion of pathogens to other plants [4] (Additional file 1: Figure 1a and b).

Although described primarily as a plant pathogenic organism, before invading the plant tissues, *Xac*, as well as other bacteria from the same genus, can develop as an epiphyte or saprophytic organism, which is capable of surviving outside the plant [5, 6]. Under the impact of several abiotic factors, which already demonstrates its adaptive capacity to adverse conditions [7], *Xac* invades tissue of compatible plant hosts becoming a phytobacterium. Likewise, so that it can survive within the plant tissues, *Xac* is adapted to stressful conditions imposed by the plant in the early periods of infection [8] and expresses genes related to pathogenicity and virulence [9].

The response to the invading organism may vary among plants depending on which plant-microorganism recognition and immunity pathway is triggered: the Effector-Triggered Immunity (ETI) or the Pathogen-Associated Molecular Pattern-Triggered Immunity (PAMP-PTI) [10] (Additional file 1: Figure 1c). The highly conserved N-terminal domain of flagellin (flg22), for example, is characterized as a plant bacterial PAMP [11, 12]. Lipopolysaccharides (LPS) and structural or secreted proteins also act as PAMPs [13]. The Effector-Triggered Immunity (ETI) pathway may occur in plants carrying the plant’s resistance protein (R), which recognizes the pathogen’s avirulence protein (Avr) [14]. The PAMP-PTI pathway triggers the expression of genes related to defense [15], which may occur by a Mitogen-Activated Protein Kinase (MAPK) cascade or by production of Reactive Oxygen Species (ROS) [16], culminating in regulation of the activity of genes involved in plant defense [17]. However, this process of molecular plant-pathogen recognition and interaction is highly dynamic that some proteins secreted by the pathogen inhibit the cascade effect induced by PAMPs, modulating host response process against plant pathogen attack [18].

When the pathogen presence is detected the production of ROS is the earliest plant cell response, and the oxidative stress generated under this condition is a fundamental process called Plants` Oxidative Burst (POB) [19]. During the POB response, species such as superoxide anion radical (O_2_^●-^), hydrogen peroxide (H_2_O_2_), hydroxyl radical (OH^●^), and organic peroxides (R-OOH) are massively produced [16]. These compounds are very reactive, causing modifications in biomolecules such as DNA, RNA, proteins, lipids and their precursors, which cause defective cell function, including mutations and bacterial replication blockage and death [20]. Thus, if the invading microorganism is susceptible to ROS, adaptation inside the plant does not take place, and the microorganism dies due to cell lysis or inability to replicate. However, some microorganisms are capable to metabolize and/or induce inactivation of the ROS function, resulting in plant tissue colonization and disease induction.

Several previous studies have shown that certain *Xanthomonas* proteins are involved in the adaptation to stress conditions [21–25]. Our group has been contributed to this knowledge by using a qualitative MudPIT strategy (Multidimensional Protein Identification Technology - protein chromatography followed by mass spectrometry) [26]. In that previously work it was shown that several differentially expressed proteins related to the Type II and III secretion systems and to Type IV pilus are key factors in initial stages of *Citrus sinensis* infection by *Xac*. Here our aim was to expand on the results of [26] by adopting a quantitative proteomic approach, coupling 2D gel technique to MALDI-TOF/TOF analysis. Our experimental set-up was designed to investigate the sequence of events related to adaptation of *Xac* during POB in response to *Xac* infection in its compatible host, *Citrus sinensis* (L. Osbeck). We compared the protein profile in the early stages of infection (3 and 5DAI) with the profile under non-infectious conditions (NB medium) and with a medium that mimics plant conditions (XAM1), both for 24 h of growth. This comparative analysis identified a set of 124 differentially abundant proteins (up- and down-regulated). Through this analysis, it was possible to get a better understanding of *Xac*’s adaptive process when in contact with the defense resources imposed by the plant during infection.

## Results and discussion

**Fig. 1:**
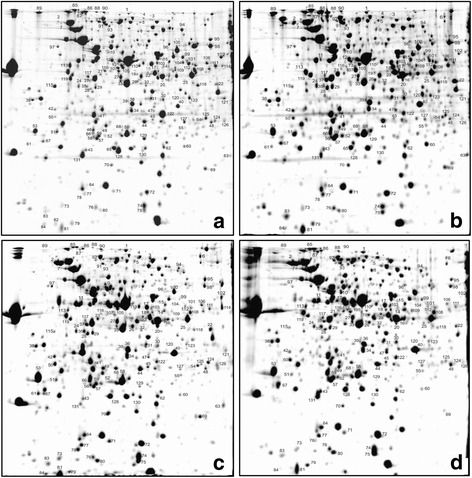
Representative 2D proteome images of the *Xac* proteins. **a** *Xac* grown in nutrient broth (NB) medium. **b** *Xac* grown in inducing virulence medium (XAM1) for 24 h, simulating 1DAI in plant. **c** *Xac* exudates from host citrus plants at 3DAI. **d** *Xac* exudates from host citrus plants at 5DAI. All samples were separated in 18 cm IPG strips across a linear pH range (4–7) using IEF in the first dimension and 12.5% SDS-PAGE in the second. Gels were stained with Coomassie *blue*. Numbers indicate the protein spots identified by mass spectrometry analysis (Tables 1). All experiments were done in triplicate

Most of our results are based on 2D gel maps of *Xac*. The reference 2D gel maps of four protein extracts from *Xac* separated by 2D SDS-PAGE are shown in Fig. 1 (panels A to D). Panel A illustrates a representative 2D gel (pH 4–7 and 10–90 kDa) with separation of proteins from *Xac* grown in NB medium (control condition). Panel B shows a representative 2D gel of proteins from *Xac* grown in XAM1, a medium without any plant-derived molecules and which induces in vitro expression of factors related to pathogenicity and virulence [27, 28]. This condition simulates 1DAI. Henceforth, references to 1DAI are used to mean expression levels observed for *Xac* growing in XAM1. Panels C and D show 2D gel maps obtained from proteins extracted from citrus leaves at 3DAI and 5DAI.

**Fig. 2:**
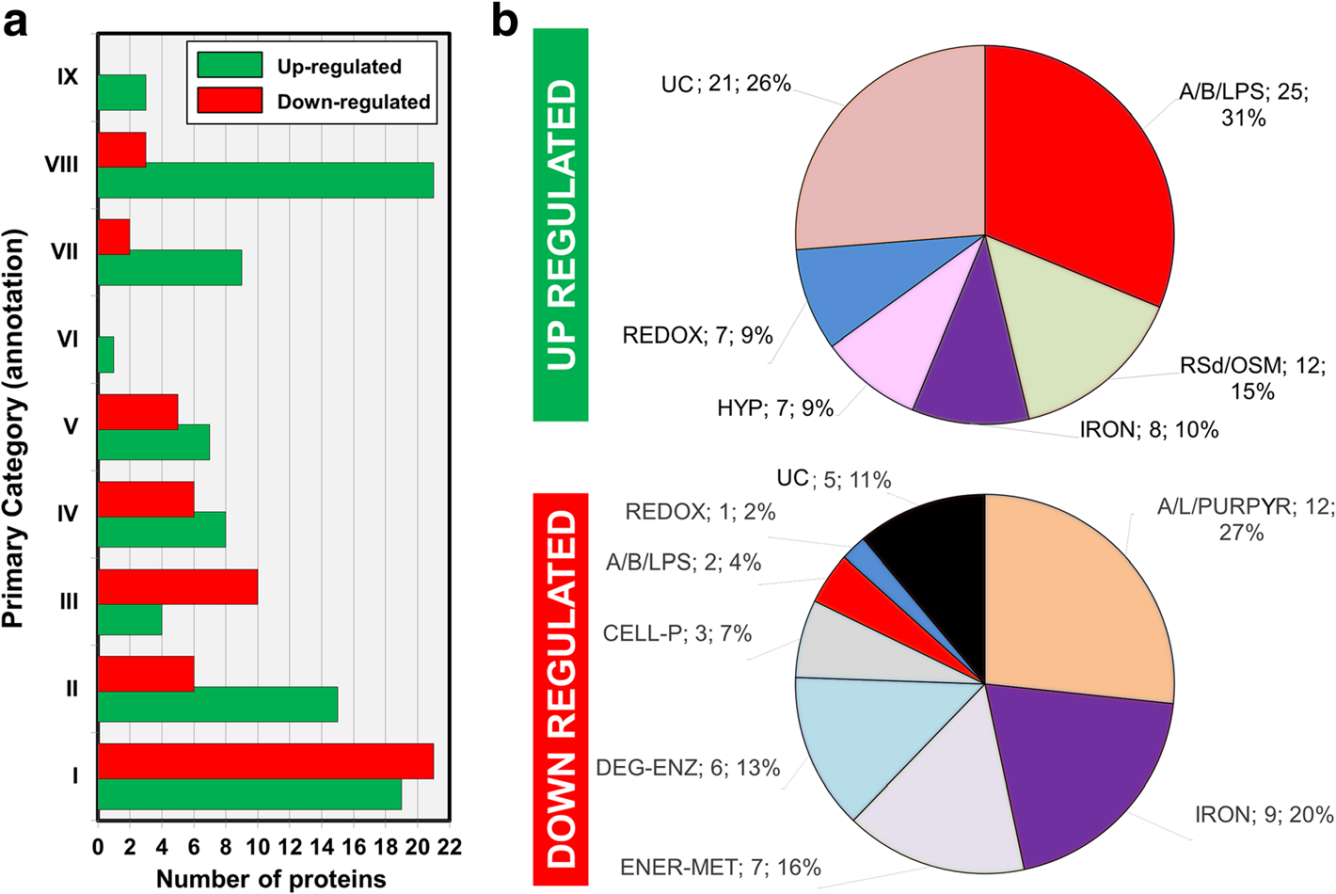
Comparative analysis of functional protein profile of *Xac* under different conditions. **a** Comparison of differentially abundant proteins (up-regulated is shown in *green* and down-regulated in *red*) according to the categories used in the genome annotation [131]: I - Intermediary metabolism, II - Biosynthesis of small molecules, III - Macromolecule metabolism, IV - Cell structure, V - Cellular processes, VI - Mobile genetic elements, VII - Pathogenicity, virulence, and adaptation, VIII - Hypothetical, IX - ORFs with undefined category. **b** Differentially abundant proteins according to status of expression in infectious conditions and the number of proteins in each category. Three of these categories (A/B/LPS, IRON and REDOX) are common in both analyses. For details, check Table 1. Categories: A/B/LPS - Adhesion, biofilm, and LPS modulation; ROSd/OSM - Reactive species depletion and osmotic control; IRON - iron acquisition and metabolism; HYP - Hypothetical proteins; REDOX - Reduction and oxidation related proteins; UC - Uncharacterized class; A/L/PURPYR - Amino acids, lipids, purines and pyrimidines metabolism; ENER-MET - Energy metabolism; DEG-ENZ - Degrading enzymes; CELL-P - Cellular process

The image data analysis of triplicate 2D gels of each extract shows about 600 spots representing proteins. From the 600 spots, 220 were differentially abundant while under infectious conditions. Those spots whose expression value was greater than 1.5-fold (or 1.5×) relative to the control (as are all expression values mentioned from here on) were excised from the gel and identified by MALDI-TOF-MS/MS and database searching leading to the identification of 168 proteins in 157 spots. Among them, 124 spots with unique identification and statistically significant variation in intensity were detected: 79 were up-regulated proteins and 45 correspond to down-regulated proteins (Additional file 2). A comparative analysis of up-regulated proteins (green bars) and down-regulated proteins (red bars) in infectious conditions, classified by functional categories, is shown in Fig. 2a. Sixty-three out of the seventy-nine up-regulated proteins (~80%) were manually grouped into six functional categories (the remaining proteins do not have an associated function). Likewise, 40 out of the 45 down-regulated proteins (89%) were manually grouped into eight functional categories (Fig. 2b).

**Fig. 3:**
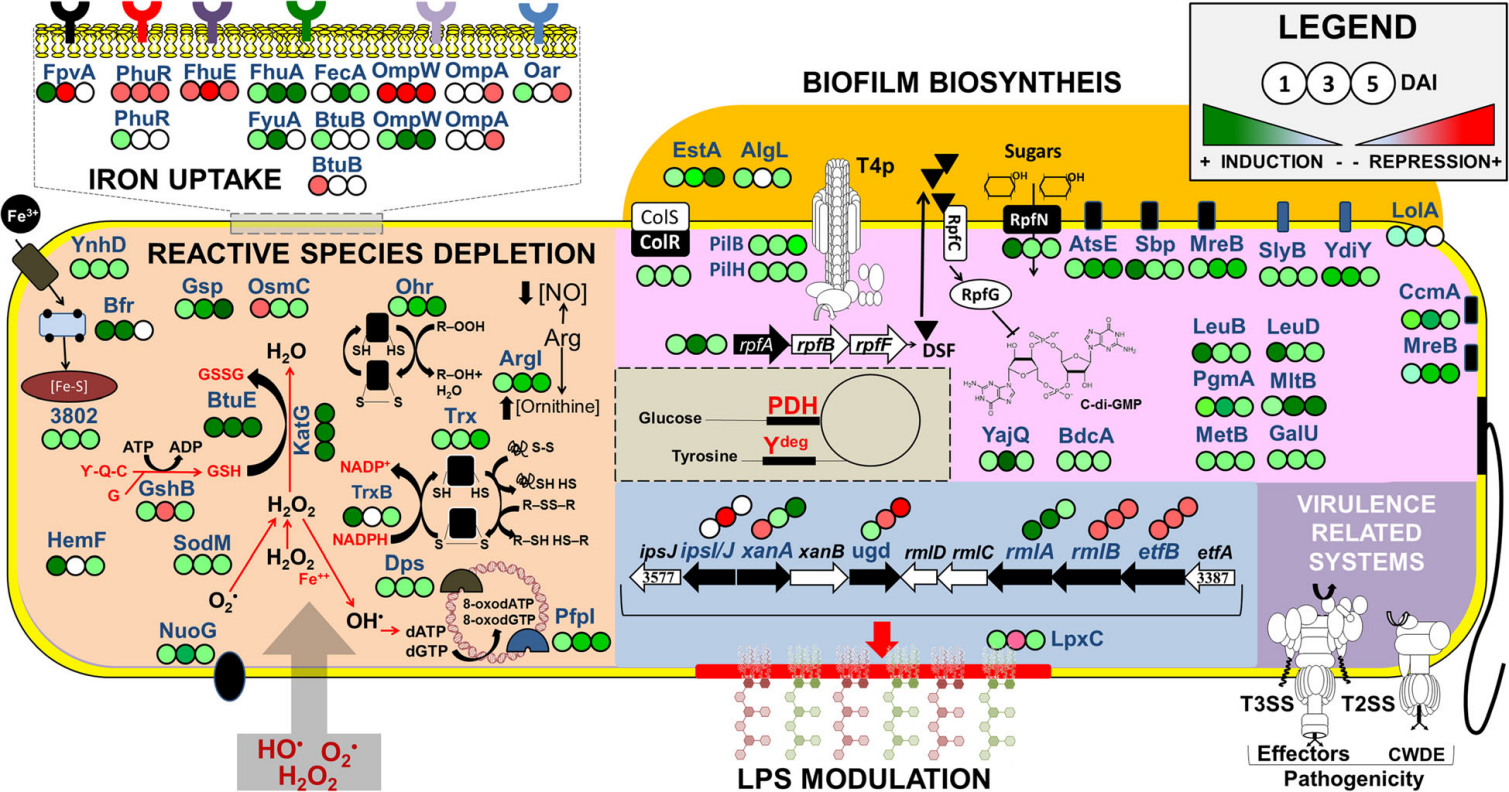
Functional overview of differential expression proteins in infection condition. The order of *circles* and colors represent the day where they were detected and expression level, respectively (see legend)

Some of these categorizations are summarized in Tables 1, 2, and 3, and an integrated illustration representing 62 differentially abundant proteins is shown in Fig.3. In what follows, we discuss these results in detail.

**Table 1:** Differential expression of proteins in infectious conditions related to RS depletion, osmotic control, oxi-nitroreductases, and iron acquisition and metabolism

SPOT	PROTEIN	Product	Gene name	Expression	XAM1/NB	3DAI/NB	5DAI/NB	CLASS
2	XAC2830	TonB-dependent receptor	*fhuA*	Up	62.06	144.00	148.00	IRON
3	XAC4368	TonB-dependent receptor	*fecA*	Up	0.00	8.99	3.16	IRON
4	XAC1301	Catalase	*katG*	Up	11.52	19.27	35.31	RSd/OSM
5	XAC3201	TonB-dependent receptor	*fyuA*	Up	2.57	1.56	0.48	IRON
6	XAC2698	NADH-ubiquinone oxidoreductase, NQO3 subunit	*nuoG*	Up	5.21	13.89	1.78	REDOX
7	XAC2829	Outer membrane hemin receptor	*phuR*	Up	1.48	156.00	158.00	IRON
20	XAC1434	Nitrous oxidase accessory protein NosD, contains tandem CASH domains	*nosD*	Up	13.37	54.21	13.10	REDOX
25	XAC4349^a^	Bifunctional oxireductase/alginate lyase	*algL*	Up	2.79	0.12	3.43	REDOX
26	XAC0339	Oxidoreductase	*----*	Up	4.23	17.89	10.42	REDOX
28	XAC0288	Oxidoreductase	*mocA*	Up	42.40	27.05	10.87	REDOX
30	XAC4109	Aerobic coproporphyrinogen III oxidase	*hemF*	Up	3.23	0.24	1.70	RSd/OSM
31	XAC2005	Thioredoxin reductase	*trxB*	Up	1.90	0.99	2.29	RSd/OSM
32	XAC3103	Glutathione synthetase	*gshB*	Up	1.09	0.61	2.75	RSd/OSM
35	XAC4009	Arginase	*argI*	Up	1.34	8.41	11.58	RSd/OSM
38	XAC2783	Thioredoxin	*trx*	Up	2.93	1.89	2.42	RSd/OSM
45	XAC2936	ABC transporter ATP-binding protein	*ynhD*	Up	1.04	4.60	4.37	IRON
48	XAC1160	Oxidoreductase	*bdcA*	Up	0.33	0.31	2.58	REDOX
53	XAC3664	Outer membrane protein	*ompW*	Up	2.28	4.08	3.36	IRON
58	XAC2386	Superoxidase dismutase	*sodM*	Up	1.45	1.84	1.75	RSd/OSM
60	XAC0554	Nitroreductase	*----*	Up	22.22	0.56	0.53	REDOX
61	XAC1149	Bacterioferritin	*bfr*	Up	23.71	155.00	0.00	IRON
62	XAC3123	Starvation-inducible DNA-binding protein	*dps*	Up	2.07	2.70	3.04	IRON
64	XAC2369	General stress protein	*gsp*	Up	6.00	88.95	117.14	RSd/OSM
66	XAC4346	Glutathione peroxidase	*btuE*	Up	24.25	1.73	13.30	RSd/OSM
67	XAC2932	Protease	*pfpI*	Up	6.09	11.82	14.43	RSd/OSM
74	XAC2915	Osmotically inducible protein	*osmC*	Up	0.95	2.89	2.89	RSd/OSM
75	XAC0282	Organic hydroperoxide resistance protein	*ohr*	Up	5.30	19.40	24.36	RSd/OSM
85	XAC2743	Oar protein	*oar*	Down	1.81	0.34	0.72	IRON
86	XAC4274	OmpA-related protein	*----*	Down	1.66	0.27	0.43	IRON
87	XAC4273	OmpA-related protein	*----*	Down	1.69	0.05	0.88	IRON
88	XAC2742	TonB-dependent receptor	*btuB*	Down	0.00	0.00	0.05	IRON
89	XAC3444	TonB-dependent receptor	*btuB*	Down	1.78	0.00	0.00	IRON
91	XAC0823	Outer membrane hemin receptor	*phuR*	Down	1.17	0.00	0.00	IRON
92	XAC0176	Ferripyoverdine receptor	*fpvA*	Down	0.00	0.09	1.20	IRON
93	XAC3498	Outer membrane receptor for ferric iron uptake	*fhuE*	Down	0.40	0.05	0.40	IRON
111	XAC3802	Fe-S oxidoreductase, related to NifB/MoaA family	*----*	Down	0.12	0.15	0.07	REDOX
128	XAC3354	Outer membrane protein W	*ompW*	Down	0.00	0.00	0.00	IRON

^a^Also present at A/B/LPS Class

**Table 2:** Differential expression of proteins in infectious conditions related to adhesion, biofilm, and LPS biosynthesis

SPOT	PROTEIN	Product	Gene name	Expression	XAM1/NB	3DAI/NB	5DAI/NB	CLASS
1	XAC1882	Aconitase	*rpfA*	Up	5.71	6.92	7.59	A/B/LPS
8	XAC3239	Pilus biogenesis protein	*pilB*	Up	1.48	4.33	9.39	A/B/LPS
9	XAC3300	Lipase	*estA*	Up	1.93	21.98	96.64	A/B/LPS
11	XAC3579	Phosphoglucomutase	*xanA*	Up	0.19	3.91	7.29	A/B/LPS
13	XACb0007 XAC3225	Lytic mureintransglycosylase Transglycosylase	*mlt* *mltB*	Up	8.11	71.06	113.00	A/B/LPS
17	XAC3602	Cystathionine gamma-lyase-like protein	*metB*	Up	0.00	2.67	12.91	A/B/LPS
18	XAC2504	Regulator of pathogenicity factors	*rpfN*	Up	354.75	6.19	13.17	A/B/LPS
21	XAC3456	3-isopropylmalate dehydrogenase	*leuB*	Up	43.75	7.62	18.57	A/B/LPS
22	XAC1017	ABC transporter sulfate binding protein	*sbp*	Up	45.65	13.96	1.78	A/B/LPS
23	XAC0656	Rod shape-determining protein	*mreB*	Up	0.96	3.08	8.57	A/B/LPS
25	XAC4349^a^	Bifunctional oxireductase/alginate lyase	*algL*	Up	2.79	0.12	3.43	A/B/LPS
33	XAC0785	UDP-3-O-[3-hydroxymyristoyl] N-acetylglucosamine deacetylase	*lpxC*	Up	1.33	0.27	2.48	A/B/LPS
34	XAC3584	Glucose-1-phosphate thymidylyltransferase	*rmlA*	Up	1.74	2.14	2.33	A/B/LPS
36	XAC2292	UTP-glucose-1-phosphate uridylyltransferase	*galU*	Up	1.49	1.80	1.57	A/B/LPS
37	XAC4219	Lipid-binding SYLF domain	*----*	Up	30.72	20.18	8.09	A/B/LPS
40	XAC3966	Outer membrane lipoprotein SlyB	*slyB*	Up	2.45	3.17	4.10	A/B/LPS
42	XAC0623	Putative salt-induced outer membrane protein YdiY	*ydiY*	Up	6.83	8.70	1.38	A/B/LPS
43	XAC0190	Uncharacterized lipoprotein	*----*	Up	2.21	1.30	4.24	A/B/LPS
44	XAC0834	Two-component system regulatory protein	*colR*	Up	1.58	2.01	2.50	A/B/LPS
49	XAC3457	3-isopropylmalate dehydratase small subunit	*leuD*	Up	65.18	2.46	8.27	A/B/LPS
54	XAC1028	Phosphoglycerate mutase	*pgmA*	Up	3.46	6.88	0.77	A/B/LPS
68	XAC1344	Cytoskeletal protein CcmA, bactofilin family	*ccmA*	Up	1.89	9.49	8.66	A/B/LPS
77	XAC0108	Atse	*atsE*	Up	9.99	22.76	27.21	A/B/LPS
78	XAC1154	Regulatory protein pilH family	*pilH*	Up	_	1.00	1.00	A/B/LPS
81	XAC3671*	Uncharacterized conserved protein YajQ, UPF0234 family	*yajQ*	Up	8.74	84.52	0.19	A/B/LPS
123	XAC1717	2-dehydro-3-deoxyphosphooctonate aldolase	*kdsA*	Down	0.51	0.06	0.07	A/B/LPS
129	XAC2008	Outer-membrane lipoproteins carrier protein precursor	*lolA*	Down	0.08	0.05	0.00	A/B/LPS

* putative hypothetical gene manually reannotated

^a^Also present in REDOX Class

**Table 3:** Expression profile of proteins in infectious conditions related to amino acids, lipids, purine-pyrimidine, and energy metabolism

Spot	PROTEIN	Product	Gene name	XAM1/NB	3DAI/NB	5DAI/NB	CLASS
90	XAC1885	Aconitate hydratase 2	*acnB*	0.02	0.50	0.03	ENER-MET
99	XAC0454	Homogentisate 1,2-dioxygenase	*hmgA*	0.14	0.00	0.17	A/L/PURPIR
100	XAC1533	Dihydrolipoamide dehydrogenase	*ldp*	0.98	0.61	0.95	ENER-MET
101	XAC3388	Citrate synthase	*gltA*	1.74	0.06	0.28	ENER-MET
103	XAC3688	D-amino acid dehydrogenase subunit	*dadA*	0.00	0.09	1.39	A/L/PURPIR
108	XAC2012	3-ketoacyl-coa thiolase	*fadA*	0.78	0.49	0.86	A/L/PURPIR
109	XAC0265	Acyl-coa dehydrogenase	*acdA*	0.00	0.00	0.00	A/L/PURPIR
113	XAC0452	4-hydroxyphenylpyruvate dioxygenase	*----*	0.00	0.13	0.43	A/L/PURPIR
114	XAC1348	Acetoacetyl-coathiolase	*atoB*	0.12	0.04	0.08	A/L/PURPIR
115	XAC3609	Fumarylacetoacetate hydrolase	*uptA*	0.07	0.04	0.04	A/L/PURPIR
116	XAC0445	Pyruvate dehydrogenase E1 beta subunit	*pdhB*	0.13	0.10	0.10	ENER-MET
117	XAC0902	Transaldolase B	*talB*	0.22	0.13	0.62	ENER-MET
118	XAC2916	Aspartate carbamoyltransferase	*pyrB*	0.62	0.08	0.06	A/L/PURPIR
120	XAC2502	1-phosphofructokinase (fructose 1-phosphate kinase)	*fruK*	300.00	0.00	0.00	ENER-MET
121	XAC2715	Acetyl-coenzyme A carboxylase carboxyl transferase	*accD*	0.20	0.06	0.00	A/L/PURPIR
122	XAC2547	Dihydrodipicolinate synthetase	*dapA*	0.19	0.05	0.14	A/L/PURPIR
124	XAC1314	Enoyl-coa hydratase	*paaF*	0.24	0.09	0.35	A/L/PURPIR
125	XAC3586	Electron transfer flavoprotein beta subunit	*etfB*	0.73	0.05	0.09	ENER-MET
127	XAC3578	IpsJ protein	*ipsI/ipsJ*	0.21	0.08	0.16	A/L/PURPIR

### Xac adaptation during interaction with plant tissue

In this section, we present results for proteins that play a role in adaptation of *Xac* through Reactive Species depletion and osmotic adaptation (ROSd/OSM), Oxide-Nitroreductases (REDOX), and iron acquisition and metabolism related proteins (IRON) that can contribute to its survival within plant tissues (Table 1). Together, the proteins related with this category comprise 10 of the 45 down-regulated proteins (22%) and 27 of 79 up-regulated proteins (33%), which emphasize the importance of these proteins in the adaptive profile of *Xac* after 24 h of infection.

#### Reactive oxygen species depletion

KatG, SodM, GshB, Trx, TrxB, and BtuE proteins are directly involved in the metabolism and depletion of ROS, reducing the structural damage of bacterial DNA, proteins and lipids. The importance of these enzymes in the adaptive process of *Xanthomonas*, avoiding programmed cell death for example, has been previously described [21–23, 29]. We observed that SodM and KatG were up-regulated in all experimental conditions. However, the expression of KatG increases progressively and reaches a peak value (35×) at 5DAI. In this context, it is worthwhile to point out that recent studies have shown that mutation in *katG* gene (XAC1301) leads to a massive reduction of virulence in *Xanthomonas* [24]. This gene is also involved in tumor development induced by *Agrobacterium tumefaciens* in *Kalanchoe* plants [30] and has been described as a key component in *Erwinia crysanthemi* virulence induction process [31]. Additionally, thioredoxin (Trx) and thioredoxin reductase (TrxB), together with glutathione-glutaredoxin, which control ROSs concentration by reducing disulfide bonds [32], showed differential expression levels. These values (0.99 to 2.93 times up) were relatively low when compared to those of other analyzed proteins, but the values remained relatively stable from 1DAI to 5DAI. Glutathione synthetase (GshB) and glutathione peroxidase (BtuE) were also up-regulated while BtuE showed high levels of expression at 1DAI (24×) and 5DAI (13×).

#### Osmotic adaptation and oxide-Nitroreductases

Concerning the mechanism of organic peroxides, Ohr and OsmC proteins are part of the depletion of this mechanism in bacteria. Ohr, primarily described in *Xanthomonas*, is expressed in the presence of organic peroxides, but not under the influence of osmotic stress [33–36], while OsmC is induced only by osmotic stress and is controlled by a variety of regulators responsive to stress [37]. Our results demonstrate that OsmC has lower expression than Ohr in all conditions, but both increase over time, with Ohr reaching 24× at 5DAI. It is possible that the later expression of OsmC reflects not only the condition to which *Xac* is being exposed, but also the formation of alcohols resulting from Ohr catalysis [37]. Gsp protein was also detected as being up-regulated. In other organisms Gsp has a key role in response to stress induced by tert-butyl hydroperoxide (tBOOH - the organic peroxide), heat shock, acid pH, detergents (bile salts, SDS), ethanol, sodium chloride, and H_2_O_2_ [38, 39].

Regarding oxidoreductases and nitroreductases proteins, up-regulation of eight oxidoreductases and one nitroreductase in the same conditions were showed in our findings and also suggests a direct relationship between iron homeostasis and stress [40]. Among oxidoreductases, MocA showed increasing expression under the conditions studied reaching the peak value of 42× at 1DAI, the same expression profile of a nitroreductase coded by XAC0554 that was up-regulated 22× at 1DAI. The proteins encoded by XAC0339, XAC2698 (NuoG), and XAC1434 (NosD) reached peaks at 3DAI (17.9×, 13.9×, and 54,21× respectively) while the protein encoded by XAC3802 (Fe-S oxidoreductase) was down-regulated in all stages of infection. Among the proteins annotated as having REDOX function, the most interesting is BdcA, encoded by the gene XAC1160. Although it has well conserved short chain dehydrogenase domain, the orthologous protein of BdcA in *E. coli* was characterized as Novel c-di-GMP-binding protein for biofilm dispersal [41]. Structurally, the protein presents a Rossmann nucleotide binding domain, hence the fact that it was annotated as an oxidoreductase, but unlike other proteins involved in binding and catalysis, c-di-GMP does not have the classic domains previously described for this function [42]. In this context, BdcA could also have function involved in signaling mediated by c-di-GMP, which is important in adaptive processes of *Xac* inside the plant.

#### Iron acquisition, internalization, and metabolism

In addition to the above-mentioned proteins directly related to ROS depletion, proteins related to iron storage were seen to be also up-regulated. Bacteria synthesize proteins that are capable of storing iron under stress conditions to avoid Fenton reactions, controlling ROS generation [43]. This is the case for proteins Bfr and Dps, differentially abundant in all experimental conditions. While Dps showed differential expression similar to most proteins associated with ROS depletion, Bfr showed a large differential expression value at 1DAI (23×), with a peak of 155× at 3DAI, and then back to non-differentially expression levels at 5DAI. In fact, Dps has been characterized as a protein capable of storing about 500 atoms of Fe^3+^ in its central cavity [44], whereas Bfr is able to store about 1800 atoms of Fe^3+^, even though it binds to DNA [45]. Dps is also induced under carbon-limiting conditions or by the action of ROS being able to bind DNA to protect it from the action of oxidizing agents [46]. Under physiological conditions, in the absence of ROS, and in conditions of iron deficiency, these proteins help to maintain iron homeostasis. Thus, these proteins may reduce the rate of Fe^2+^ in the cellular environment, allowing the survival of *Xac* inside the plant [40].

Iron internalization proteins were found up-regulated in our infectious conditions. In an environment with high oxidative potential and high activity of Bfr and Dps in storing Fe^3+^, increasing internalization of this metal may compensate the lack of soluble Fe. This may explain the regulation of four distinct TonB-dependent receptors (TBDRs) possibly related to iron acquisition (FhuA, FyuA, PhuR, OmpW, and FecA) and one iron-regulated ABC transporter protein (YnhD). Except for FecA, up-regulated only in direct contact with the plant at 3DAI and 5DAI, all other TBDRs were up-regulated also at 1DAI. PhuR, FhuA, and OmpW achieved expression peaks at 3DAI (156×, 144×, and 4.8× respectively), maintaining this level of expression also at 5DAI. Interestingly, while these six TBDRs were up-regulated, nine other proteins with related functions were down-regulated under the same conditions (FpvA, PhuR, BtuB_2x_, Oar, OmpW, FhuE, and OmpA_2x_).

It is important to highlight that although TBDRs are classically related to internalization and iron metabolism, these proteins have been described as having some other functions such as internalization of carbohydrates [47]. Moreover, some TBDRs of *X. campestris* carrying a Carbohydrate Utilization Locus (CUT) are responsible for acquisition of carbohydrates derived from plants [48]. For *Xac*, a TonB-dependent transducer has been described as responsible for regulating pathogenicity-related genes [49] (Additional file 3). These receptors might be able to bind to the same compound, but in different physiological conditions. In fact, no siderophore produced by *Xac* has been fully characterized yet, although biochemical tests have suggested its production [50]. Furthermore, a comparative proteomic study using 2D gels carried out on *Xac* [51] mature biofilm and planktonic cells showed that different TBDRs and OMPs are up- or down-regulated depending on the lifestyle of the organism, as observed for this work. The involvement of these proteins in the process and characterization of biofilm formation is well known. It is possible, therefore, that all of these TBDRs and OMPs that were shown here to be up- and down-regulated are also associated with cell adhesion and biofilm formation in conjunction with other proteins detected with this function in *Xac* during the infectious process (described below).

#### Metabolism of related proteins

Finally, some related proteins, as Argl that correspond to an arginase that protects *Helicobacter pylori* against acid stress, were explored in our discussion besides modulating colonization in mice. Arginase activity may also reduce the pool of arginine that could be converted to NO^•^ (nitric oxide) + citrulline by iNOS (inducible Nitric Oxide Synthase) [52]. Nitric oxide is a modulator of defense response in plants [53]. In our experiments, ArgI showed increased expression in all the experimental conditions analyzed, with a peak of 12× at 5DAI. Thus, it is possible that the increased expression of this protein in infectious condition could reduce the stress generated by the plant through reduction of NO^•^, a hypothesis previously put forward in a comparison of a virulent strain of *Xylella fastidiosa* (9a5c) with another non-virulent strain (J1A12) [54]. In that study, the authors suggested that the presence of this gene in the virulent strain could explain in part its success in colonizing the xylem tissue since the gene could reduce the plant defense response, which is different from what is observed in the non-virulent strain not carrying this gene. In addition, HemF and PfpI stood out in our results as proteins associated with virulence and adaptation in stressful conditions. HemF is an aerobic coproporphyrinogen III oxidase, an enzyme responsible for the synthesis of protoporphyrin, which is a precursor in the synthesis of heme. Although up-regulated in all infectious conditions, HemF reached peak expression at 1DAI (3.2×). In *E. coli*, synthesis of heme is observed when cytochrome and catalase syntheses occur [55]. HemF is one of the enzymes that require molecular oxygen to catalyze their reaction [56]. This protein was shown to be a member of the oxidative stress-induced regulon responsible for protecting cells from oxidative damage [57], which could explain why it has differential expression together with other proteins related to ROS adaptation, as described above. Similarly, the protease PfpI was up-regulated in all conditions with peak expression of 11× at 5DAI. PfpI is a member of DJ-1/ThiJ/PfpI superfamily, and plays a role in DNA protection under non-stress conditions. In a *Pseudomonas aeruginosa pfpI-*mutant strain*,* a dramatic increase of H_2_O_2_-induced damage was observed, besides biofilm formation changes [58]. Although not classically related to ROSd or REDOX function, ArgI, HemF, and PfpI seem to be crucial during the infectious process in response to POB, and the loss of its functions could cause a disturbance in the adaptive process that would culminate in reducing virulence phenotypes.

### Plant immune system evasion and protection against stress induced by plant

The proteins grouped under adhesion, biofilm, and LPS synthesis (A/B/LPS) add to 25 (31,6%) out of the 79 up-regulated proteins and only 2 (5%) out of the 45 down-regulated proteins in infectious conditions, and are listed in Table 2.

#### LPS synthesis and biofilm during virulence process

Bacterial LPS and EPS are well described as being involved in plant pathogenesis [59]. LPS is the main component of the outer membrane in gram-negative bacteria, whose primary function is to maintain cell integrity against external agents. LPS is also fundamental in bacterial ecology for its role in mediating adhesion between bacteria or between bacteria and plant tissue. Besides acting as pathogen-associated molecular patterns (PAMPs), LPS constitutes the first layer in plant innate immunity induction and is referred to PAMP-triggered immunity in plants [60]. LPS is structurally divided into four parts: Lipid A, inner core, outer core, and O-antigen. Lipid A represents a hydrophobic moiety facing the outside of the outer membrane, allowing the LPS structure to be anchored, and is considered essential for growth and virulence of bacteria. Mutations in genes related to Lipid A synthesis are considered lethal, as it drastically alter the structure of this key component of membrane organization [61].

One of the differentially abundant proteins in this category was LpxC (a metalloprotease), involved with Lipid A biosynthesis. Some studies have focused on the use of inhibitors of LpxC and other proteins related to LPS biosynthesis as potential new antibiotics [62]. However, in the context reported in this paper, an up-regulation of this protein in infectious conditions (specially 5DAI) could be related to modulation of LPS structure in an attempt to ensure protection against bacterial stress-induced reduction during PTI response [63], as was described in *P. aeruginosa* [64].

Some of the genes involved in the synthesis of LPS and O-antigen are inserted in tandem in a single genomic region in *Xac* genome. This region can be represented by *ipsJI-xanAB-ugd-rmlDCBA-etfBA-8CCSG-4XacSG-wzt-wzm-metB* [65], where CCSG stands for Citrus Canker Specific Gene, which are genes found only in *Xac*, X. fuscans, Xac* and Xac ^*W*^ [66, 67], and *Xac* SG stands for *Xac* specific genes. One of the CCSGs is *nlxA*, and it has recently been characterized in *Xac* as essential for EPS and LPS synthesis, motility, biofilm formation, and resistance in the presence of peroxides during redox imbalance [68]. This emphasizes the importance of this whole region in the biology of *Xac*. RmlA is up-regulated in all experimental conditions [1DAI (1.7×), 3DAI (2.1×), and 5DAI (2.3×)]. This gene is inserted into the *rmlDCBA*cluster involved in O-antigen, which is important in structuring the bacterial cell as well as protecting against external action. In *Stenotrophomonas maltophilia* from Xanthomonadaceae family, mutations in the genes *rmlA*, *rmlC*, and *xanB* lead to a biofilm formation that presents differentiated characteristics [69]. It is noteworthy that *rmlB*, *ugd*, and *ipsJI*, which are found in this large genome region are down-regulated in infectious conditions. This could be related to expression modulation of these proteins in early stages of infection analyzed in this study. MetB, encoded by the last gene of the cluster, corresponds to a cystathionine gamma-synthase protein [66, 70, 71]. In *X. oryzae*, *metB* mutant showed colony morphologies of relatively reduced mucoidy, implying reduction of EPS productivity, beyond a loss of O-antigen [72]. Furthermore, MetB presents a central role in cysteine, homocysteine, and methionine metabolism, which is essential for bacterial growth. Consequently, MetB has been identified as a potential drug target [73]. Finally, XanA (phosphoglucomutase) were up-regulated (3DAI 3.9× and 5DAI 7.3X) in infectious conditions. Together with XanB, both proteins have been previously reported as essential to the process of xanthan gum synthesis [74], being responsible for the production of monomers of carbohydrates such as trehalose, which can play the role of an osmoprotectant [26].

Recently, Guo and colleagues found that GalU (XAC2292) is essential to the formation of EPS and LPS in *Xac*, and that biofilm formation is reduced in the *galU* mutant strain [75, 76]. Moreover, in *Pseudomonas syringae* this gene may change pathogen-host interaction, inducing PTI in tomato and preventing the survival of the pathogen inside the plant [77]. This is consistent with our finding that this protein is up-regulated in infectious conditions.

Alginate lyase (coded by XAC4349) catalyzes the degradation of alginate, a complex copolymer of α-L-guluronate, and its C5 epimer β-D-mannuronate was up-regulated at 1DAI (2.8×) and 5DAI (3.4×). The alginate structure protects LPS structure against stress in marine brown algae, *Azotobacter vinelandii*, *Azotobacter chroococcum*, and in several species of *Pseudomonas* [78–80]. Considering that the function of alginate lyase in *P. aeruginosa* is to detach cells from the surface, so they may spread and colonize new sites [81], it is possible that in *Xac* this enzyme functions to avoid competition with other bacteria that produce alginate within the plant [82], leading to more susceptibility to the action of this stressful condition imposed by plant during infection. Alternatively, alginate lyase may modulate the structure of their LPS and regulate the induction of plant innate immunity [83]. Thus, like AtsE, a more detailed study of the functionality of alginate lyase is necessary for a better understanding of its role in the mechanism of plant-pathogen interaction.

Concerning biofilm formation, Sbp is encoded by the first gene of an operon (XAC1017–1020) associated to an ABC transporter. This protein was also up-regulated in infectious conditions, reaching 45.6× at 1DAI, and then decreasing to 13,9× and 1,8× at 3 and 5DAI respectively. When Sbp is mutated, it leads to reduced biofilm formation in *Xac* [84]. In fact, biofilm formation is a key component in the pathogenicity process of *Xac*. In *Listeria* and *Rhizhobium* a link between loss of the homolog gene and decrease in biofilm has also been shown [85, 86].

Type IV pilus (T4P) correspond to an important structure found on the surface of filamentous bacteria during adhesion, and they are involved in twitching motility and other activities such as bacterial surface adherence, biofilm formation, colonization, genetic material uptake, and virulence [87]. PilB is involved in pilus assembly (extension/polymerization) and acts as a motor responsible for twitching motility together with PilT. Mutations in PilB stop completely T4P formation [88], and a mutation in *Xylella fastidiosa* homolog gene disabled twitching and inhibited the bacteria from colonizing upstream vascular regions in plants [89]. On the other hand, the same mutation did not affect attachment to polysaccharides present in insect mouth nor foregut extracts [90]. PilH, characterized as a regulatory gene, when mutated leads to a reduced swarming and significant change in biofilm formation in *Pseudomonas aeruginosa* [91]. The differential expression of these T4P proteins under infectious conditions observed in our results combined with the results cited in the literature suggest that these proteins are probably important to colonization, displacement favored via swarming, and biofilm formation. No significant differential expression of PilH was detected. However, for PilB it was observed differential expression at 3DAI (4.3×) and 5DAI (9.4×).

MreB is a protein involved in cell shape in rod-shaped bacteria. Patch motility is largely powered by cell-wall synthesis, and MreB polymers may restrict diffusion of patch components in the membrane and orient patch motion [92]. MreB regulates production, location, and function of T4P in *Pseudomonas aeruginosa*, and is therefore fundamental to the process of biofilm-mediated colonization [93]. This may be linked with the concomitant differential expression of PilB mentioned above. In our study both proteins MreB and PilB have very similar expression profiles and levels suggesting that MreB may regulate PilB expression also in *Xanthomonas*.

Phosphoglycerate mutase (PgmA) participates in the bacterial energy metabolism (glycolysis and gluconeogenesis), but some studies have suggested its role in biofilm formation [94, 95]. It has also been described as critical to the process of virulence induced by other pathogens [96]. We observed PgmA to be up-regulated in all infectious conditions [1DAI (3.5×), 3DAI (6.9×), 5DAI (0.77)].

#### Adhesion and virulence induction

Regarding attachment to host cells, AtsE coded by XAC0108 is a protein required for adhesion. In *Agrobacterium tumefaciens*, AtsE is inserted in the chromosome region containing genes required for virulence and attachment to host cells [97]. Homologs occur in all genera belonging to Xanthomonadaceae family except in *Xylella* (data not shown).

During infection, the virulence induction is a key process that allows tissue colonization. The *rpfA* gene is involved in synthesis and cell signaling mediated by diffusible signal factor (DSF), which modulates *quorum sensing* in bacteria from Xanthomonadaceae family. Mutation in the regulator of pathogenicity factors genes (*rpfA-I*) decrease virulence of such bacteria [98]. Furthermore, RpfA acts as an aconitase and is therefore essential for iron homeostasis [99, 100]. In our experiments, RpfA was up-regulated in all experimental conditions reaching a peak of differential expression at 5DAI (7×). By contrasting the period of RpfA expression, it is possible to highlight the RpfN protein, which showed the highest peak of expression at 1DAI (350×), reducing to 6.12× and 13.17× in later times respectively. RpfN is a sugar-selective porin in *X. oryzae* (Xoo) according to UniProt/EMBL [101]. Mutation in *rpfN* increases synthesis of polygalacturonate lyase [99], which induces degradation of plant cell wall. In *Burkholderia pseudomallei*, OprB protein, homologous to RpfN, was shown to be important for biofilm formation [102], and more recently RpfN has been shown to be up-regulated under physiological conditions when *Xac* biofilm is produced [51]. These findings suggest that increasing synthesis of degradation enzymes may increase the supply of substrates in contact with plant tissue, compensating the lack of sugar caused by the mutation.

The gene encoding Mlt (XACb0007), a transglycosylase protein, is located in the pXAC64 plasmid in a region of 7260 bp. A gene with 95% of sequence similarity (*mltB* – XAC3225) can be found in the chromosome. This region in the plasmid is flanked downstream by *xopE2*, while the same region on the chromosome is flanked upstream by *xopE3* and downstream by *xopA1* [66]. All these Xop proteins are known to be T3SS effectors [103] and therefore directly related to virulence induction in *Xanthomonas*. Interestingly, *mlt/mltB* show similarity to *hopA1* (a T3SS helper protein), which contributes to translocation of T3SS effectors [104]. Mutation in the chromosomal copy of this gene led to a complete loss of *Xac* virulence in its compatible host [105]. More recently, Ferreira and co-authors [106] showed that the plasmid copy of *mltB* gene carried by Tn3-like ISXax2 was indeed functional and required to generate symptoms in plant. The authors also showed that MltB, type III secretion system effector protein (T3SEs), and transcription activator-like effectors (TALEs) are strictly related to the emergence of virulence and pathogenesis. Furthermore, it was also demonstrated that the transposase ISXax2 and associated mobile insertion cassettes (MICs) probably act as key agents in spread and modulation of bacterial virulence and host-specificity in this group of plant pathogens. Thus, up-regulation of Mlt/MltB in infectious conditions appears to have a direct correlation with effector secretion and virulence induction, which could validate its function based on sequence similarity with hopA1. It is important to highlight that this was the protein that showed the highest differential expression, increasing along the infectious process: 1DAI (8×), 3DAI (71×), and 5DAI (113×).

EstA is an esterase described as fundamental to the process of virulence of some pathogens, being essential to promote colonization of host tissue [107]; and it was also up-regulated, with 6× at 1DAI and 97× at 5DAI. In *P. aeruginosa,* this lipase binds to alginate increasing enzyme stability and accumulation of lipase in EPS [79]. Mutation in this gene in *X. oryzae* led to reduced virulence of the pathogen in rice [71], and this proteins has been described as enzyme targeted for biochemical identification of pathogenic strains of *Xanthomonas* [108].

ColR and ColS act together as a two-component regulatory system, representing the regulatory and sensing units respectively. In *Xac,* there are three copies of this system (XAC0834–35, XAC1221–22, and XAC3250–49). Recently, studies have revealed the importance of this dual component system in pathogenicity of *Xac* [109], as well as a component required for virulence and growth in iron-limiting conditions in *Xoo* [110]. Thus, up-regulation of ColR under infectious conditions [1DAI (1.6×), 3DAI (2×), 5DAI (2.5×)] may have two fundamental roles: one as a protein that participates in biofilm synthesis, and the other as a protein that takes part in the Fe deficiency-induced oxidative condition imposed by plants.

LeuB and LeuD, encoded by part of a gene cluster (*leuABDC*), are involved in valine, leucine, and isoleucine biosynthesis. They were up-regulated in all conditions, but it was on conditions that simulates 1DAI that their expressions were highlighted, 43× and 65× respectively to LeuB and LeuD. Mutation in *leuB* in *Xac* led to reduced *Xac* virulence in the compatible host [105]. Likewise, mutation in *leuD* in *Pseudomonas savastanoi* pv. *savastanoi* resulted in massive virulence reduction in *Olea europaea* plants [111]. Thus, this whole cluster may play a critical role in the process of plant-pathogen interaction.

Although it has been previously annotated as a hypothetical protein, the protein encoded by the gene XAC3671 actually corresponds to YajQ protein. In a recent study in *X. campestris* it was demonstrated that YajQ belongs to a new class of effector binding proteins of c-di-GMP which contributes to virulence induction [112]. Finally, five other proteins previously annotated as hypothetical were included in this functional category after analysis of its COGs and reannotation; all of them were up-regulated during the infectious process. XAC3966 and XAC0190 encode membrane lipoproteins with similar expression profiles. XAC0623 is a salt-induced putative outer membrane protein (YdiY) with expression peak at 1DAI (6,83×) while XAC4219 corresponds to a protein with a lipid-binding SYLF domain. The last and most interesting is the protein encoded by XAC1344, which corresponds to a cytoeskeletal protein (CcmA). These proteins are associated with cell motility, cell morphology, and cell division since they are members of bactofilin family [113]. In *M.xanthus,* bactofilin BacP interacts with PilB and PilT (previously described and up-regulated), which are responsible for extension and retraction of T4p respectively, and thus for the motility of the cell [114]. In a comparative proteome study between wild type strain and a mutant strain for *hrpB* gene, Zimaro and collaborators demonstrated that CcmA is one of the proteins suppressed in the mutant strain evidencing a possible relationship between type III secretion system and motility in *Xac* [51], as previously reported in other model organisms [115].

### Energy metabolism and metabolism of amino acids, lipids, and purine-pyrimidine

The energetic metabolism is a crucial process to ATP and NADH/NADPH generation. Under specific conditions, as in case of redox imbalance, some metabolic alterations are important for bacterial fitness, as it is for the antioxidant process maintenance. Our results indicate that some enzymes related to glycolysis, transport chain reaction, and oxidative phosphorylation are up-regulated (Additional file 4: Figure S2, Table 3).

On the other hand, TCA cycle proteins were down-regulated, in particular the crucial enzymes for pyruvate conversion to Acetyl-CoA. Some recent results have shown that pyruvate secretion and TCA cycle deregulation are crucial for pathogenicity in some bacteria [116], which is consistent with our findings [117]. However, in *Xac* such connections have not been shown until now.

Interestingly, our results also indicate that three out of five proteins that participate in tyrosine conversion to fumarate were down-regulated: 4-hydroxyphenylpyruvate dioxygenase (XAC0452), HmgA - homogentisate 1,2-dioxygenase (XAC0454), and UptA – fumaryl acetoacetate hydrolase (XAC3609) (Additional file 4). While investigating the protein expression profile of *P. aeruginosa* in the presence of PQS (*Pseudomonas* Quinolone Signal), Bredenbruch and colleagues observed repression of two homologous proteins (PA2008-FahA and PA2009-HmgA) with concomitant up-regulation of a series of proteins involved in redox process response [118]. This is an indication that oxidative stress and metabolism of tyrosine have some functional relationship. The role of PQS has also been investigated in cell-cell communication, *quorum sensing*, and iron entrapment to the balance for life and death decisions in *Pseudomonas aeruginosa* populations [119–121].

We observed that citrate synthase was down-regulated at 3DAI (0.06×) and 5DAI (0.28×). Even though it has been shown that citrate synthase is necessary for optimum levels of biofilm formation and virulence in *Burkholderia cenocepacia* [122], this protein could have been down-regulated by the absence of the substrate Acetyl-CoA, formed in the previous step by pyruvate dehydrogenase complex in *Xac*. For analogous reasons aconitase could also be suppressed in this case. It is important to note that aconitase have a Fe-S prosthetic group, and synthesis of this protein may be limited by oxidative conditions to which it is exposed.

### Hypothetical proteins

In addition to eight hypothetical proteins that were reannotated, manually categorized and described in the previous sections (XAC3802, 1434, 1344, 4219, 3966, 0623, 0190, and 3671), five other proteins were reannotated based on COGs and included in the category of proteins with undefined category (UC): XAC0007, a putative Zn-dependent protease; XAC0193, a phosphohistidine phosphatase; XAC0381, a ketosteroid isomerase; XAC3140, a periplasmic TolA-binding protein; and XAC3851, an uncharacterized mettaloenzyme (YdcJ). All of them were up-regulated in infectious conditions. Thus, only seven proteins remained in the group of hypothetical proteins up-regulated in infectious conditions: XAC1093, 1364, 2246, 2315, 3680, 3866, and 3981 (Additional file 5).

**Fig. 4:**
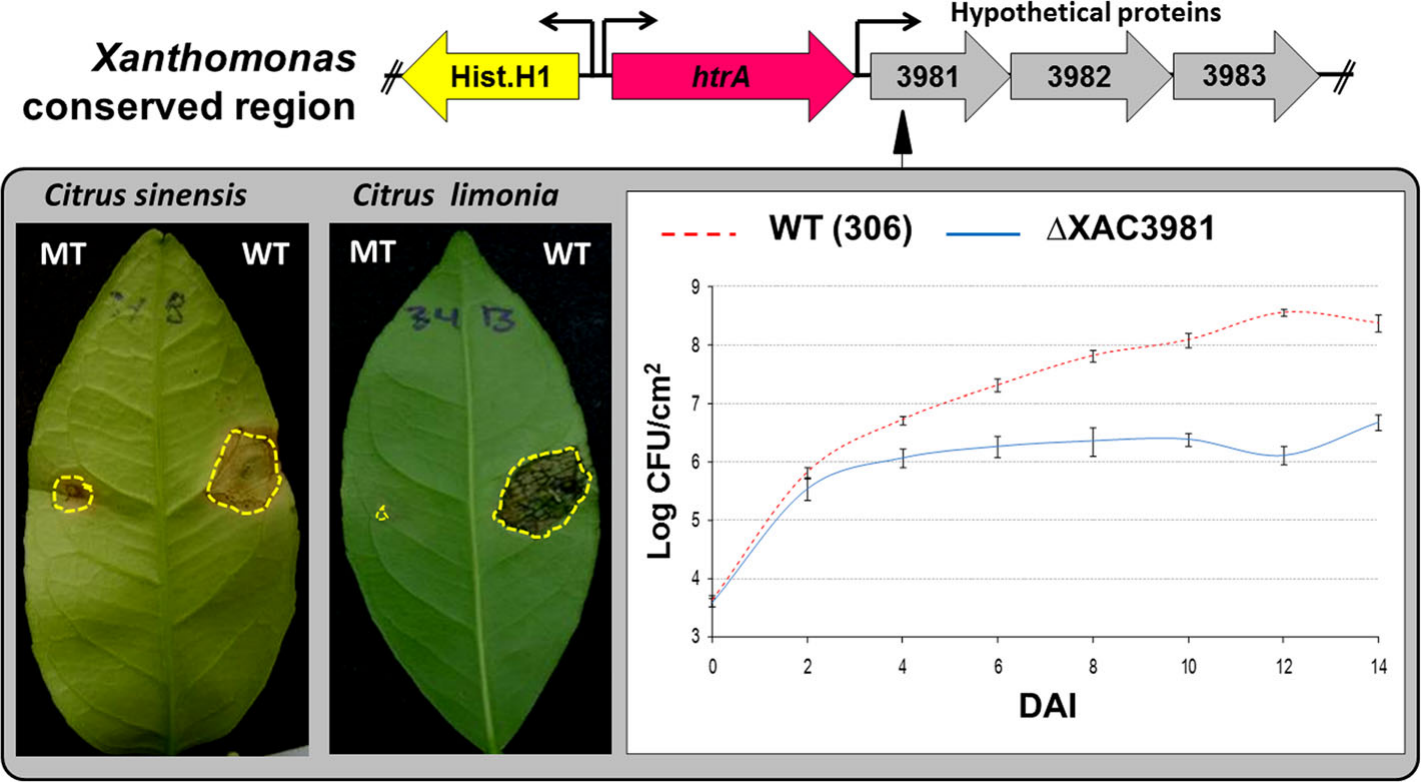
Analysis of the phenotypic profile of virulence in the mutant XAC3981, located in a putative operon with XAC3982 and XAC3983 genes (*gray arrows*). The mutant XAC3981 led to a marked reduction in the virulence phenotype in both *Citrus sinensis* and *Citrus limonia* after 14DAI. The growth curve of the mutant in vivo showed less bacterial titration along the infectious process, which could explain the absence of observed phenotypes. This mutated gene is flanked by *htrA* (upstream), and by two hypothetical proteins (downstream) whose orthologous pairs were found only in bacteria of c (See Additional file 6: Figure S4)

In an attempt to understand the possible role of these hypothetical proteins in *Xac* metabolism, a clone library containing more than 10,000 *Xac* mutants previously described was analyzed in an attempt to find a mutant for these hypothetical genes that would reduce the virulence in *Citrus* plants [105]. Only one of these mutants (ΔXAC3981) did not seem to cause any symptoms throughout the infectious process (14 days of observation when inoculated in compatible hosts (*Citrus sinensis* and *Citrus limonia*) (Fig. 4). In vivo growth curve of bacteria showed that this gene appears to be essential for the adaptive process of *Xac* during the infectious process. The Tn-insertion in XAC3981 likely disrupts expression of XAC3982 and XAC3983 since these genes are predicted to form an operon [123]. Although the colony count curves are still valid, we cannot affirm that the mutation of XAC3981 is responsible for virulence reduction phenotype, but rather the combined function of the operon.

The gene *htrA* encoding a protease DO (XAC3980) is located upstream of gene XAC3981, whose mutation results in complete absence of symptoms [105]. In addition, there are two hypothetical proteins (XAC3982–3983) downstream of XAC3981, which together with XAC3981 are found only in bacteria from Xanthomonadaceae family (Additional file 6), and in the same order (i.e. syntenic) according to STRING [124]. In *X. oryzae* strains this region appears as a single operon according to OperonDB [123], and it is adjacent to a downstream group of genes encoding Hrp proteins, more specifically a HrpF peninsula [125].

Although it cannot be classified as a putative pathogenicity island since does not show any classic sign of being a product of horizontal gene transfer, this region may have been selected in a recent ancestor of Xanthomonadaceae and seems to be essential in maintaining the virulence process in bacteria of the genus *Xanthomonas*.

### An overview of the intricate adaptive mechanism of *Xac* inside plant

**Fig. 5:**
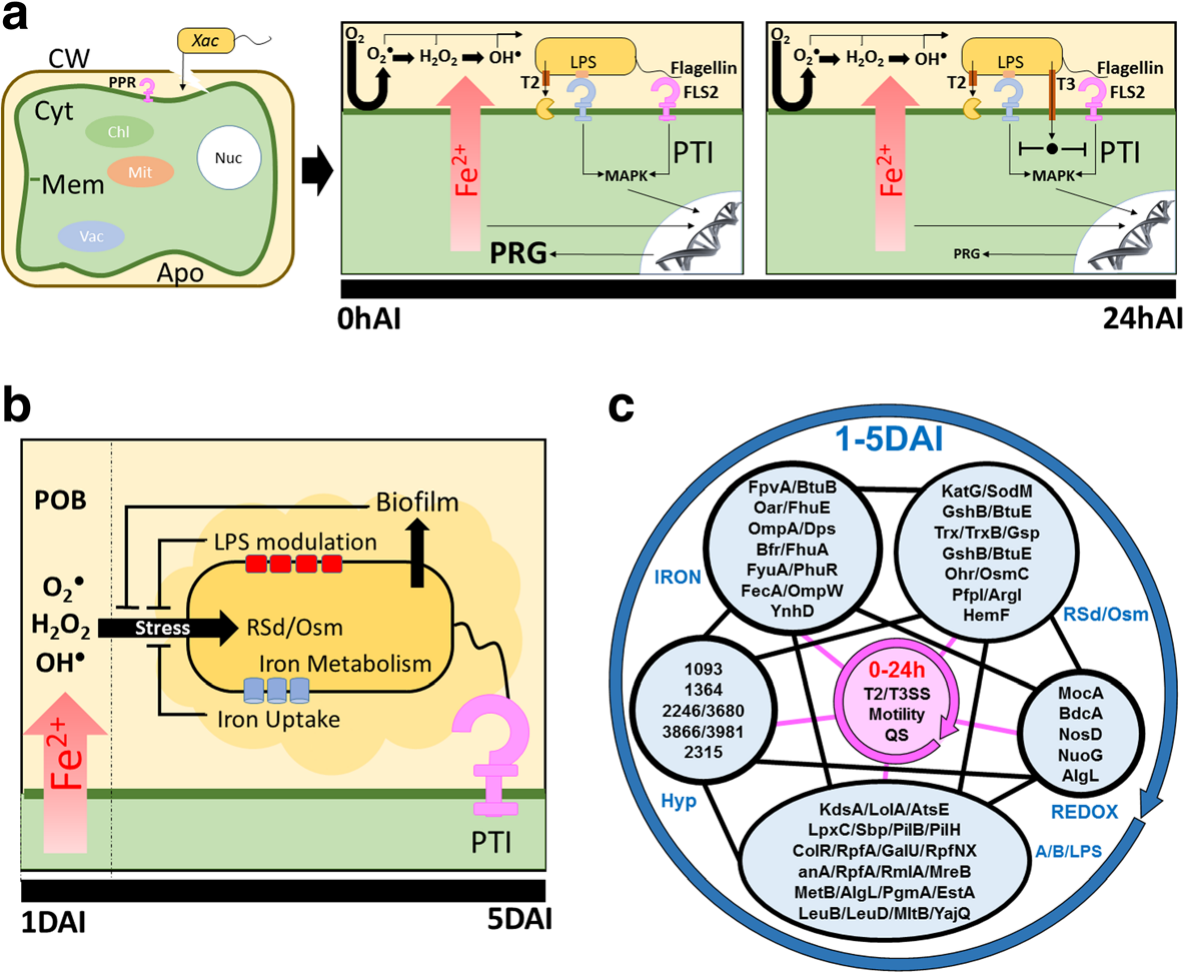
Integrated outlook of proteins secreted between 1-5DAI. **a** During the first hours of infection, secretion system structural proteins, and proteins related with motility and *quorum sensing* are secreted. After recognition of PAMPs, a cascade of reactions culminates in the activation of PTI in order to avoid penetration of bacteria inside plant tissues increasing the ROS production during POB. Subsequently, *Xac* secretes effector proteins to overcome plant defenses and promote bacterial virulence. These processes may occur between 0 and 24 h after infection. **b** After 24 h of infection, *Xac* secretes higher amounts of proteins related with reactive species depletion and osmotic adaptation (ROSd/OSM), iron uptake and metabolism, biofilm formation, and LPS modulation in order to protect themselves against the stress caused by POB. **c** After activation of virulence related proteins during the first 24 h (pink), a protein repertoire involved in ROS depletion, EPS biosynthesis and LPS modulation, iron uptake and metabolism, and biofilm formation is active and working in an integrated manner between 1 and 5 DAI

Here we sketch a proposed scenario for the first five days of *Xac* infection based on the above results (Fig. 5). In the very first hours of infection, secretion system structural genes and effector genes are activated and the secreted proteins act on plant tissue, in order to overcome plant defenses and promote bacterial virulence [126] (Fig. 5a). At this point, we would also expect that proteins related to flagellum-depended motility and *quorum sensing* are also acting, which was not captured by our experiments because our observations started 24 h after infection. Once the plant detects the presence of the pathogen through specific PAMP receptors, the defense mechanisms are activated beginning massive production of oxygen reactive species such as superoxide (Fig. 5a) in POB. At this time, the infection could be controlled, or in case of compatible host the secondary defense response is induced by iron intracellular redistribution that promote the transcription of genes related to H_2_O_2_ production [127]. This in turn induces in *Xac* the expression of proteins directly related to ROS depletion, osmotic control, biofilm production, and iron acquisition and storage (Fig. 5b). At the same time, the pathogen modulates LPS synthesis through two different mechanisms. Lipid A modification is responsible for reducing the permeability of the outer membrane proteins to antimicrobial proteins, decreasing resistance responses from the host. In contrast, modification or loss of O-antigen leads to delay in recognition by the plant, allowing intracellular survival and protection against oxidative stress [128]. Together with LPS modulation, the expression of proteins related to adhesion, including OMP, and biofilm formation would increase protection against defenses by the plant [129, 130]. Although the differentially abundant proteins were divided into distinct functional groups, our results suggest that this intricate network of proteins could be important in the adaptive process and defense against POB induced by host plants after 24 h of infection (Fig. 5c). The efficiency of all these processes described above would enable the pathogen to rapidly colonize, replicate, and disperse from the initial infection site to other tissues of the same plant or even to other citrus host plants, effectively triggering disease spread.

## Conclusions

Our results provide the most complete repertoire of proteins active in the infectious process by *Xac* in a compatible host, adding substantially to what was previously known, in particular with respect to work published by some of us [26].We show that this repertoire is involved in ROS depletion, EPS biosynthesis and LPS modulation, iron uptake and metabolism, and biofilm formation, and that the component proteins work in an integrated manner. Previous studies have focused on classical virulence systems such as types II and III secretion systems (*rpf* genes); these systems are important for the pathogen to be able to start the infectious process, but they cannot ensure infection success by themselves. For the infection to be sustained other sets of proteins need to come into play, and these sets of encoded proteins were identified in this study. These proteins are primarily related to adaptation (defense) and damage to plant tissue (attack). Even though adaptation and induction of damage to plant tissue may seem unrelated biological processes, we believe that they are synergistic. Thus, the set of proteins identified in our study is associated with evasion of plant immune system increasing the antimicrobial resistance through protection against host defenses, improvement of intracellular survival, and protection against oxidative burst. In addition, through the expression of proteins related with iron uptake and metabolism, biofilm formation, and depletion of reactive species *Xac* reduces the production of reactive species by the plant while intensifying production of biofilm and proteins related with reactive species depletion. Therefore, the proteins identified in our study are potential biotechnological targets for disease control since they seem to be essential for adaptation and survival inside plant tissues.

## Methods

### Bacteria and culture conditions

The *Xac* strain 306 pathotype A [131] used in this study was obtained from the culture collection of plant pathogenic bacteria at IAPAR (Instituto Agronômico do Paraná, PR, Brazil). This strain was grown at 28 °C in NB medium (Difco™ NB – 5 g/L peptone and 3 g/L meat extract) with shaking at 200 rpm for 24 h, and was grown at 28 °C in XAM1 medium (7.4 mM (NH_4_)_2_SO_4_; 16 mM KH_2_PO_4_; 30 mM K_2_HPO_4_; 1.6 mM sodium citrate (C_6_H_5_Na_3_O_7_.2H_2_O); 10 mM fructose; 10 mM sucrose; 1 mg/mL BSA; pH 5.4) with shaking at 200 rpm for 24 h. The cells were analyzed by spectrophotometer in 600 nm (OD_600_) at an optical density corresponding to the log phase (1.0).

### Plant inoculation

‘Pêra Rio’ orange trees (*Citrus sinensis* L. Osbeck) were grown in a greenhouse for 4–6 months. *Xac* inoculum was prepared by cultivating the bacteria in solid NB medium at 28 °C for 16 h in order to obtain cellular mass. The cells grew to an optical density of OD_600nm_ = 1.0, and then diluted with sterile distilled water to OD_600nm_ = 0.3 (10^8^ CFU/mL). The suspension of cells was then used to infiltrate 50 young leaves by injection with a syringe without the needle, directly into the apoplastic space on the abaxial face of the leaves.

### Bacteria recovery from inoculated leaves

*Xac* recovery was performed as described by Mehta and Rosato [132], with some modifications. The citrus leaves were excised from plants at 3 or 5DAI. The leaves were rinsed with 70% alcohol, sliced into small pieces using a sterile razor blade, and maintained for 20 min under agitation in sterile glass Becker containing 400 mL of cold (4 °C) distilled water. After the incubation period, the exudate was centrifuged at 10,000 x*g* for 10 min at 4 °C and the pellet containing the exudated *Xac* was used for protein extraction.

It is important to emphasize that 1DAI correspond to the bacteria cultivated in XAM1 media and 3 or 5DAI inoculated in plant.

### Protein extraction

The extraction of total proteins was performed as described by Mehta and Rosato [132], with some modifications. Each *Xac* pellet was washed in phosphate buffer (1.24 g/L K_2_HPO_4_; 0.39 g/L KH_2_PO_4_; 8.8 g/L NaCl, pH 7.2). Subsequently, the pellet was suspended in 0.75 mL of extraction buffer (0.7 M sucrose; 0.5 M Tris-HCl, pH 7; 30 mM HCl; 50 mM EDTA; 0.1 M KCl, 40 mM DTT) and incubated for 15 min at room temperature. The same volume of phenol was added. After 15 min of agitation, the suspension was centrifuged at 10,000 x*g* and 4 °C for 3 min, and the supernatant was recovered. This procedure was repeated twice. The proteins were precipitated with five volumes of 0.1 M ammonium acetate in methanol, and the precipitate was washed once with 80% acetone. Protein concentrations were estimated by the Bradford method (Bio-Rad, Hercules, CA, USA).

### 2D–gel electrophoresis

The precipitates (1 mg from each sample) were solubilized in 250 μL of sample buffer (8 M urea, 2% CHAPS, 70 mM DTT, 0.001% bromophenol blue-BPB, 0.5% ampholytes of pH 4–7). First dimension was carried out in 13 cm strips (pH 4–7 linear gradient) in the IPG-Phor unit as described by the manufacturer (GE Healthcare). Precast IPG strip was focused in three steps (500 V for 1 h; 1000 V for 1 h; 8000 V, 16,000 Vh). The temperature was maintained at 20 °C. After isoelectric focusing, each strip was incubated for 12 min in 10 mL of 50 mM Tris-HCl buffer pH 6.8, 6 M urea, 30% *v*/v glycerol, 2% *w*/*v* SDS, 2% DTT, followed by a second incubation step in the same buffer solution, except that DTT was replaced by 2.5% iodoacetamide. Strips were then rinsed in Tris-glycine electrode buffer, transferred to homogeneous 12.5% SDS-PAGE and overlaid with 0.5% *w*/*v* agarose in running buffer containing BPB. Gels were run in a Hoefer SE600 system at 70 V for 10 min and 25 mA/gel for 7–8 h. A molecular mass marker ladder Mark12™ (Invitrogen) was used. The gels were fixed with 40% ethanol and 10% acetic acid for 30 min and then stained with Coomassie Blue R 250 (Sigma-Aldrich), 0.025% Coomassie Brilliant Blue, 40% ethanol, 10% acetic acid, for 90 min. All experiments were done in triplicate and first and second dimension gels were electrophoresed under identical conditions.

### Image and data analysis

Direct scanning and image analysis was performed by MELANIE 2D gel analysis software (version 7.05). All experiments were done in triplicate to ensure reproducibility. Spots were quantified on the basis of their relative volume, that is, the spot volume divided by the total volume over the whole set of gel spots. The protein spots were manually confirmed. A 1.5-fold change was set as criterion: only those spots with change in abundance of more than 1.5-fold were taken into account. Differentially abundant protein spots were subjected to in-gel tryptic digestion and identified by mass spectrometry.

### In-gel digestion

Protein spots were manually excised from the gels and washed with 25 mM ammonium bicarbonate in 50% acetonitrile overnight at room temperature to destain the proteins. The gel pieces were then dehydrated in 100% acetonitrile for 10 min and fully dried in a Speed-Vac centrifuge (Savant, Minnesota, USA). Gel fragments were allowed to reswell in 10 μL of the digestion buffer containing trypsin (Promega, modified sequencing grade) at a final concentration 10 ng/μL in 25 mM ammonium bicarbonate. The gel fragments were digested with trypsin for 20 h at 37 °C. The resulting tryptic peptides were extracted from the gel pieces by incubating with 50 μL of 50% acetonitrile in 5% trifluoroacetic acid twice for 15 min, first with agitation and then with sonication. Supernatants were transferred, pooled, and concentrated to near dryness in a Speed-Vac centrifuge. Each sample was then diluted with 10 μL of Milli-Q water in 0.1% trifluoroacetic acid.

### MALDI-TOF/TOF analysis

Roughly 0.4 μL of the solution of extracted peptides was mixed with an equal volume of CHCA matrix solution (10 mg/ml α-cyano-4-hydroxycinnamic acid (Aldrich, Milwaukee, WI) in 50% acetonitrile/0.1% trifluoroacetic acid) and left to dry. Data for protein identifications were obtained from 4700 Proteomics Analyzer (Applied Biosystems, Foster City, CA). Both MS and MS/MS data were acquired with a neodymium-doped yttrium aluminum garnet (Nd:YAG) laser with a 200-Hz repetition rate. Typically, 1600 shots were accumulated for spectra in MS mode while 2400 shots were accumulated for spectra in MS/MS mode. MS and MS/MS mass spectra were acquired in reflector mode and internally calibrated with trypsin autolysis peptides. Up to eight of the most intense ion signals with signal-to-noise ratio above 30 were selected as precursors for MS/MS acquisition. External calibration in MS mode was performed using a mixture of four peptides: des-Arg1-Bradykinin (m/z = 904.468), angiotensin I (m/z 1296.685), Glu1-fibrinopeptide B (m/z 1570.677), and ACTH (18–39) (m/z 2465.199). MS/MS spectra were externally calibrated using known fragment ion masses observed in the MS/MS spectrum of angiotensin I. MS/MS spectra were searched against the *Xac* genome data bank file downloaded from the NCBI, with 108,856 sequences; 41,308,596 residues entries.

The mass spectrometry proteomics data have been deposited to the ProteomeXchange via MassIVE dataset submission workflow with the dataset identifier MSV000080041.

### Database search

All data were processed using the Data Explorer Software (Applied Biosystems, CA). Proteins were identified by correlation of tandem mass spectra and *Xac* genome data bank available at NCBI, using the MASCOT™ software (Matrix Science, version 2.1). One missed cleavage per peptide was allowed and an initial mass tolerance of 0.05 Da was used in all searches. Cysteines were assumed to be carbamidomethylated, and variable modification of methionine (oxidation) was allowed. To evaluate the false positive rate of this approach, a reversed sequence databank (a database in which the sequences have been reversed) containing the same number of proteins as in the *Xac* database was constructed. Identification was considered positive if it matched at least one unique peptide.

### Determination of differentially regulated proteins

Proteins loaded on gels were normalized between replicates to partially quantify spot intensities and to minimize analytical variation among gels. To analyze protein intensity, triplicate 2D gels of the infected conditions were compared to control gels as well as to each other. At least 4 well-defined landmarks were used for matching gels: spots were quantified on the basis of their relative volume; spots a greater than 1.5-fold change in their normalized volume between two sample groups were submitted for statistical analysis; spots that exhibited a statistically significant difference were selected for mass spectrometry identification, as well as those pertaining exclusively to one group (Additional file 7).

### Comparative genome/metabolism profile and hypothetical proteins reannotation

BLAST [133] analysis was performed against the other *Xanthomonas* genomic sequences in the NCBI database to classify the hypothetical proteins differentially abundant. Metabolic pathways were obtained from KEGG (Kyoto Encyclopedia of Genes and Genomes) annotation database [134]. The hypothetical proteins were reannotated using information from COG database [135]. All these analyses were done by searches using the respective *locus* tag or by comparative sequences using the amino acids FASTA sequences.

### Mutant selection and *in planta* virulence phenotype analysis

Among the hypothetical genes differentially expressed in infectious conditions only one gene mutated (ΔXAC3981) was selected from the *Xac* mutant library previously generated by Laia and collaborators [105]. The cellular concentration was adjusted using _dd_H_2_O to an optical density of 0.3 at 600_nm_ (10^8^ CFU/mL). The ΔXAC3891 and wild type strain suspension was infiltrated separately in two points of the left and right abaxial side of young ‘Pera Rio’ sweet orange (*Citrus sinensis* L. Osbeck) and Rangpur lime (*C. limonia* L. Osbeck) leaves, respectively. After inoculation, the plants (in triplicate) were grown in a chamber at 28 °C with artificial light photoperiod. The development of citrus canker symptoms in host plants was evaluated every day, from the 3^rd^to the 14st day after inoculation, and the symptoms were registered by digital photographs.

### In vivo growth curve

The number of cells per leaf area was measured by means of a disk of 0.75 cm in diameter removed from inoculated leaves. The leaf disk was ground in 1 ml of 1 mM MgCl_2_ solution, and serial dilutions (10^−1^ to 10^−7^) were prepared. A 10 μL droplet of each dilution was deposited on the surface of solid TSA medium containing kanamycin. The plates were kept at 28 °C for 36 h, and isolated colonies were counted. The experiment was repeated independently three times.

## Additional files

Additional file 1: Figure S1. Overview of Disease progression and pathways involved at PII. (DOCX 736 kb)

Additional file 2: Table S1. Up and Down-regulated protein in infectious conditions. (DOCX 62 kb)

Additional file 3: Table S2. Profile of TonB receptors Up and Down regulated in infectious conditions. (DOCX 17 kb)

Additional file 4: Figure S2. Energy metabolism of Xac highlighting a set of proteins down-regulated in infectious conditions. (DOCX 783 kb)

Additional file 5: Table S3. Expression profile of Hypothetical and Conserved hypothetical proteins in infectious conditions. (DOCX 15 kb)

Additional file 6: Figure S3. Phylogenetic and String analysis of *Xanthomonas* conserved hypothetical protein related to new possible pathogenicity island. (DOCX 615 kb)

Additional file 7: Table S4. Number of matching peptides and score/confidence level. (XLSX 223 kb)

## Abbreviations

Avr: Avirulence proteins or T3SS-e
CUT: Carbohidrate Utilization Locus
DAI: Days After Inoculation or Days After Infection
DSF: Diffusible Signal Factor
EPS: Exopolysaccharides
ETI: Effector-Triggered Immunity
H_2_O_2_: Hydrogen peroxide
LPS: Lipopolysaccharydes
MAP(K): Mitogen-Associated Protein (kinase)
MudPIT: Multidimensional Protein Identification Technology
NB: Nutriente Broth
O_2_^•^: Superoxide anion radical
OH^•^: Hydroxyl radical
PAMPs: Pathogen-associated molecular pattern
PII: Plant-Innate Immunity
POB: Plants` Oxidative Burst
PTI: PAMP-Triggered Immunity
R-OOH: Organic Peroxide
ROS: Reactive Oxygen Species
T3SS: Type 3 Secretion System
T4P: Type IV pili
TBDRs: TonB-Dependent Receptors
*Xac*: *Xanthomonas citri* subsp. *citri* or *Xanthomonas axonopodis* pv. *citri*.
