# Supplementary material for: Proteomics-based identification of differentially abundant proteins reveals adaptation mechanisms of Xanthomonas citri subsp. citri during Citrus sinensis infection
Source: BMC Microbiol. 2017 Jul 11;17:155. doi: 10.1186/s12866-017-1063-x (PMC5504864; doi:10.1186/s12866-017-1063-x)
Supplement: Supplementary file 1 — Overview of Disease progression and pathways involved at PII. (DOCX 736 kb) [file 12866_2017_1063_MOESM1_ESM.docx]

Supplementary Material 1

**Proteomics-based identification of differentially abundant proteins reveals adaptation mechanisms of *Xanthomonas citri* subsp. *citri* during *Citrus sinensis* infection.**

Leandro M Moreira^1,2^, Márcia R Soares^3^, Agda P Facincani^4^, Cristiano B Ferreira^4^, Rafael M Ferreira^4^, Maria I T Ferro^4^, Fábio C Gozzo^5^, Érica B Felestrino^2^, RenataA B Assis^2^, Camila Carrião Machado Garcia^1,2^, João C Setubal^6,8^, Jesus A. Ferro^4^, Julio C.F. de Oliveira^7^


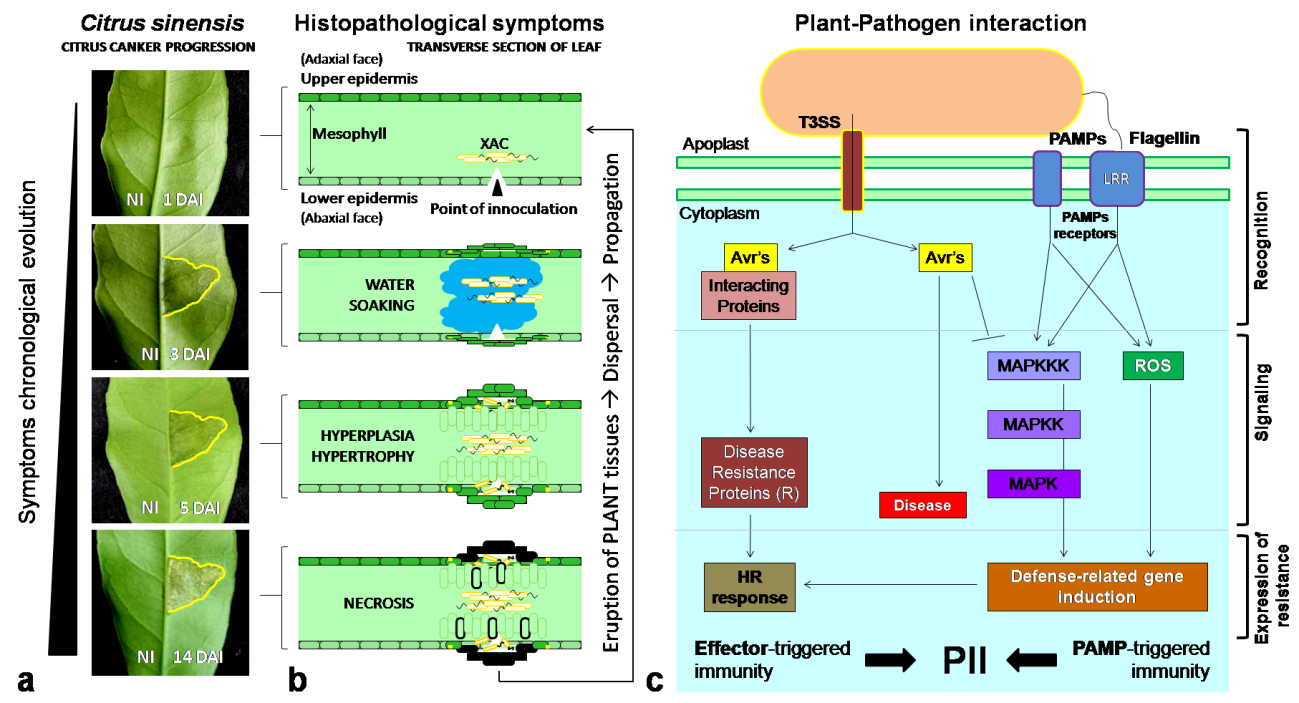


**Supplemental Figure 1. Overview of Disease progression and pathways involved at PII**. **(A)** Symptoms chronological evolution of citrus canker in *Citrus sinensis* (L.Osbeck) leaves. The yellow line borders the site of infection / spread of bacteria and highlights the progressive virulence phenotype (water soaking – 3DAI, hyperplasia/hypertrophy – 5DAI and necrosis – 14DAI). **(B)** Schematic representation depicting histopathological changes in Citrus leaf in cross section, corresponding to that seen in the pictures in A. After the 14th day, the natural infection cycle continues with consequent disruption of plant tissues and spread of *Xac*. **(C)** Schematic representation of induction of PII modulated by ETI and PTI pathways. The table in the upper right depicts the relationship of resistance/susceptibility mediated by the presence/absence and the interaction of proteins R-Avr.
