# Supplementary material for: Proteomics-based identification of differentially abundant proteins reveals adaptation mechanisms of Xanthomonas citri subsp. citri during Citrus sinensis infection
Source: BMC Microbiol. 2017 Jul 11;17:155. doi: 10.1186/s12866-017-1063-x (PMC5504864; doi:10.1186/s12866-017-1063-x)
Supplement: Supplementary file 2 — Up and Down-regulated protein in infectious conditions. (DOCX 62 kb) [file 12866_2017_1063_MOESM2_ESM.docx]

Supplementary Material 2

**Proteomics-based identification of differentially abundant proteins reveals adaptation mechanisms of *Xanthomonas citri* subsp. *citri* during *Citrus sinensis* infection.**

Leandro M Moreira^1,2^, Márcia R Soares^3^, Agda P Facincani^4^, Cristiano B Ferreira^4^, Rafael M Ferreira^4^, Maria I T Ferro^4^, Fábio C Gozzo^5^, Érica B Felestrino^2^, RenataA B Assis^2^, Camila Carrião Machado Garcia^1,2^, João C Setubal^6,8^, Jesus A. Ferro^4^, Julio C.F. de Oliveira^7^

**Supplemental Table1:** Up and Down-regulated protein in infectious conditions.

| **SPOT** | **PROTEIN** | **MW** | ***pI*** | **MW/pI 2D** | **Product (reannotationproduct)** | ***Gene Name*** | **PEP** | **XAM1/NB** | **3DAI/NB** | **5DAI/NB** | **CLASS** | **Expression** |
| --- | --- | --- | --- | --- | --- | --- | --- | --- | --- | --- | --- | --- |
| 77 | XAC0108 | 14433 | 5.06 | 18041/5.18 | Atse | *atsE* | 6 | 9.99 | 22.76 | 27.21 | A/B/LPS | up |
| 43 | XAC0190 | 28328 | 9.87 | 21870/5.27 | Conserved hypothetical protein  (Uncharacterized lipoprotein) | *XAC0190* | 5 | 2.21 | 1.30 | 4.24 | A/B/LPS | up |
| 42 | XAC0623 | 29125 | 5.21 | 27347/4.98 | Conserved hypothetical protein  (Putative salt-induced outer membrane protein YdiY) | *XAC0623* | 6 | 6.83 | 8.70 | 1.38 | A/B/LPS | up |
| 33 | XAC0785 | 34442 | 5.47 | 34862/5.47 | UDP-3-O-[3-hydroxymyristoyl] N-acetylglucosaminedeacetylase | *lpxC* | 4 | 1.33 | 0.27 | 2.48 | A/B/LPS | up |
| 22 | XAC1017 | 37842 | 6.73 | 34607/6.23 | ABC transporter sulfate binding protein | *sbp* | 8 | 45.65 | 13.96 | 1.78 | A/B/LPS | up |
| 78 | XAC1154 | 14304 | 5.16 | 17518/5.29 | Regulatoryproteinpilhfamily | *pilH* | 2 | _ | 1.00 | 1.00 | A/B/LPS | up |
| 68 | XAC1344 | 18650 | 5.45 | 23746/5.27 | Conserved hypothetical protein  (Cytoskeletal protein CcmA, bactofilin family) | *XAC1344* | 3 | 1.89 | 9.49 | 8.66 | A/B/LPS | up |
| 123 | XAC1717 | 30037 | 5.87 | 30083/6.01 | 2-dehydro-3-deoxyphosphooctonate aldolase | *kdsA* | 5 | 0.51 | 0.06 | 0.07 | A/B/LPS | down |
| 1 | XAC1882 | 99296 | 5.43 | 88533/5.55 | Aconitase | *rpfA* | 3 | 5.71 | 6.92 | 7.59 | A/B/LPS | up |
| 129 | XAC2008 | 22942 | 7.9 | 22697/5.66 | Outer-membranelipoproteinscarrierprotein precursor | *lolA* | 8 | 0.08 | 0.05 | 0.00 | A/B/LPS | down |
| 36 | XAC2292 | 32253 | 5.45 | 29036/5.55 | UTP-glucose-1-phosphate uridylyltransferase | *galU* | 8 | 1.49 | 1.80 | 1.57 | A/B/LPS | up |
| 18 | XAC2504 | 41342 | 5.98 | 40438/5.73 | Regulatorofpathogenicityfactors | *rpfN* | 6 | 354.75 | 6.19 | 13.17 | A/B/LPS | up |
| 8 | XAC3239 | 62707 | 5.56 | 65574/5.60 | Pilusbiogenesisprotein | *pilB* | 3 | 1.48 | 4.33 | 9.39 | A/B/LPS | up |
| 11 | XAC3579 | 49337 | 5.19 | 45222/5.19 | Phosphoglucomutase | *xanA* | 3 | 0.19 | 3.91 | 7.29 | A/B/LPS | up |
| 34 | XAC3584 | 34151 | 5.83 | 27727/5.57 | Glucose-1-phosphatethymidylyltransferase | *rmlA* | 6 | 1.74 | 2.14 | 2.33 | A/B/LPS | up |
| 17 | XAC3602 | 42683 | 5.68 | 41090/5.76 | Cystathionine gamma-lyase-like protein | *metB* | 4 | 0.00 | 2.67 | 12.91 | A/B/LPS | up |
| 40 | XAC3966 | 29809 | 5.83 | 28077/5.92 | Conserved hypothetical protein  (Outer membrane lipoprotein SlyB) | *XAC3966* | 8 | 2.45 | 3.17 | 4.10 | A/B/LPS | up |
| 37 | XAC4219 | 31536 | 5.82 | 34812/5.25 | Conserved hypothetical protein  (Lipid-binding SYLF domain) | *XAC4219* | 6 | 30.72 | 20.18 | 8.09 | A/B/LPS | up |
| 109 | XAC0265 | 41671 | 5.88 | 40106/5.90 | Acyl-coa dehydrogenase | *acdA* | 2 | 0.00 | 0.00 | 0.00 | A/L/PURPIR | down |
| 113 | XAC0452 | 40166 | 4.99 | 41077/5.07 | 4-hydroxyphenylpyruvatedioxygenase | *XAC0452* | 5 | 0.00 | 0.13 | 0.43 | A/L/PURPIR | down |
| 99 | XAC0454 | 50589 | 5.89 | 47136/5.96 | Homogentisate 1/2-dioxygenase | *hmgA* | 6 | 0.14 | 0.00 | 0.17 | A/L/PURPIR | down |
| 124 | XAC1314 | 28357 | 6.18 | 25550/6.19 | Enoyl-coa hydratase | *paaF* | 6 | 0.24 | 0.09 | 0.35 | A/L/PURPIR | down |
| 114 | XAC1348 | 40057 | 6.31 | 42028/6.27 | Acetoacetyl-coa thiolase | *atoB* | 2 | 0.12 | 0.04 | 0.08 | A/L/PURPIR | down |
| 108 | XAC2012 | 42104 | 6.02 | 40775/6.04 | 3-ketoacyl-coa thiolase | *fadA* | 7 | 0.78 | 0.49 | 0.86 | A/L/PURPIR | down |
| 122 | XAC2547 | 32032 | 5.7 | 26365/5.80 | Dihydrodipicolinatesynthetase | *dapA* | 8 | 0.19 | 0.05 | 0.14 | A/L/PURPIR | down |
| 121 | XAC2715 | 32390 | 6.33 | 29978/6.28 | Acetyl-coenzyme A carboxylasecarboxyltransferase | *accD* | 2 | 0.20 | 0.06 | 0.00 | A/L/PURPIR | down |
| 118 | XAC2916 | 33893 | 6.09 | 33902/6.08 | Aspartatecarbamoyltransferase | *pyrB* | 6 | 0.62 | 0.08 | 0.06 | A/L/PURPIR | down |
| 127 | XAC3578 | 25980 | 5.84 | 25221/5.97 | Ipsjprotein | *ipsI/ipsJ* | 4 | 0.21 | 0.08 | 0.16 | A/L/PURPIR | down |
| 115 | XAC3609 | 35600 | 4.8 | 33620/4.98 | Fumarylacetoacetatehydrolase | *uptA* | 5 | 0.07 | 0.04 | 0.04 | A/L/PURPIR | down |
| 103 | XAC3688 | 46931 | 5.68 | 43624/5.72 | D-aminoaciddehydrogenasesubunit | *dadA* | 6 | 0.00 | 0.09 | 1.39 | A/L/PURPIR | down |
| 97 | XAC0542 | 57131 | 5.05 | 53816/5.00 | 60kda chaperonin | *groEL* | 1 | 0.35 | 0.03 | 0.16 | CELL-P | down |
| 126 | XAC1042 | 25987 | 6.33 | 25247/6.28 | Two-component system, regulatory protein | *phoB* | 3 | 0.32 | 0.98 | 0.21 | CELL-P | down |
| 112 | XAC1523 | 40976 | 5.99 | 43257/6.01 | DnaJprotein | *dnaJ* | 8 | 0.73 | 0.03 | 0.03 | CELL-P | down |
| 95 | XAC0615 | 59242 | 6.54 | 59392/6.19 | Aminopeptidase | *XAC0615* | 6 | 0.05 | 0.87 | 0.43 | DEG-ENZ | down |
| 105 | XAC2545 | 43506 | 5.4 | 411570/5.5 | Prolinedipeptidase | *pepQ* | 8 | 0.00 | 0.00 | 0.03 | DEG-ENZ | down |
| 102 | XAC2609 | 47131 | 6.30 | 47009/6.26 | Carboxypeptidase | *XAC2609* | 7 | 0.25 | 0.03 | 0.00 | DEG-ENZ | down |
| 107 | XAC2885 | 42909 | 5.39 | 41130/5.40 | Phospholipase A1 | *XAC2885* | 5 | 0.00 | 0.03 | 0.08 | DEG-ENZ | down |
| 104 | XAC3847 | 44511 | 6.06 | 43773/5.96 | N-acyl-L-aminoacidamidohydrolase | *amaA* | 3 | 0.07 | 0.00 | 0.00 | DEG-ENZ | down |
| 96 | XAC4004 | 57739 | 5.77 | 55694/5.86 | Peptidase | *XAC4004* | 4 | 0.15 | 0.07 | 0.19 | DEG-ENZ | down |
| 116 | XAC0445 | 35587 | 5.27 | 39249/5.33 | Pyruvatedehydrogenase E1 beta subunit | *pdhB* | 4 | 0.13 | 0.10 | 0.10 | ENER-MET | down |
| 117 | XAC0902 | 34836 | 5.12 | 36490/5.20 | Transaldolase B | *talB* | 3 | 0.22 | 0.13 | 0.62 | ENER-MET | down |
| 100 | XAC1533 | 50520 | 5.80 | 51547/5.91 | Dihydrolipoamidedehydrogenase | *ldp* | 6 | 0.98 | 0.61 | 0.95 | ENER-MET | down |
| 90 | XAC1885 | 93473 | 5.22 | 90325/5.36 | Aconitatehydratase 2 | *acnB* | 2 | 0.02 | 0.50 | 0.03 | ENER-MET | down |
| 120 | XAC2502 | 33184 | 5.98 | 30014/5.94 | 1-phosphofructokinase (fructose 1-phosphate kinase) | *fruK* | 7 | 300.00 | 0.00 | 0.00 | ENER-MET | down |
| 101 | XAC3388 | 47916 | 5.97 | 45309/5.99 | Citratesynthase | *gltA* | 3 | 1.74 | 0.06 | 0.28 | ENER-MET | down |
| 125 | XAC3586 | 26449 | 6.12 | 26113/6.14 | Electron transfer flavoprotein beta subunit | *etfB* | 4 | 0.73 | 0.05 | 0.09 | ENER-MET | down |
| 76 | XAC1093 | 14516 | 5.46 | 17518/5.29 | Conservedhypotheticalprotein | *XAC1093* | 6 | 2.85 | 1.56 | 6.47 | HYP | up |
| 72 | XAC1364 | 16432 | 5.56 | 18671/5.68 | Conservedhypotheticalprotein | *XAC1364* | 5 | 2.93 | 3.48 | 5.45 | HYP | up |
| 55 | XAC2246 | 23230 | 5.97 | 24.046/5.98 | Hypotheticalprotein | *XAC2246* | 4 | 5.18 | 4.42 | 4.72 | HYP | up |
| 83 | XAC2315 | 10786 | 4.7 | 17271/4.91 | Conserved hypothetical protein  (Uncharacterized conserved protein YciI, contains a putative active-site phosphohistidine) | *XAC2315* | 2 | 8.79 | 2.48 | 1.92 | HYP | up |
| 84 | XAC3680 | 9536 | 4.85 | 17469/5.00 | Conservedhypotheticalprotein | *XAC3680* | 3 | 1.69 | 3.55 | 0.80 | HYP | up |
| 80 | XAC3866 | 13988 | 5.42 | 17380/5.33 | Conservedhypotheticalprotein | *XAC3866* | 3 | 5.33 | 54.29 | 76.17 | HYP | up |
| 82 | XAC3981 | 12172 | 4.93 | 17615/5.03 | Conservedhypotheticalprotein | *XAC3981* | 3 | 21.26 | 21.43 | 21.36 | HYP | up |
| 92 | XAC0176 | 82347 | 5.30 | 77649/5.29 | Ferripyoverdine receptor | *fpvA* | 5 | 0.00 | 0.09 | 1.20 | IRON | down |
| 91 | XAC0823 | 86172 | 6.21 | 76656/5.36 | Outermembranehemin receptor | *phuR* | 7 | 1.17 | 0.00 | 0.00 | IRON | down |
| 61 | XAC1149 | 21174 | 4.71 | 21785/4.82 | Bacterioferritin | *bfr* | 6 | 23.71 | 155.00 | 0.00 | IRON | up |
| 88 | XAC2742 | 108902 | 5.63 | 90729/5.30 | TonB-dependent receptor | *btuB* | 5 | 0.00 | 0.00 | 0.05 | IRON | down |
| 85 | XAC2743 | 111655 | 5.18 | 93622/5.13 | Oarprotein | *oar* | 8 | 1.81 | 0.34 | 0.72 | IRON | down |
| 7 | XAC2829 | 74236 | 4.89 | 76003/5.03 | Outermembranehemin receptor | *phuR* | 9 | 1.48 | 156.00 | 158.00 | IRON | up |
| 2 | XAC2830 | 86282 | 4.97 | 78152/5.01 | TonB-dependent receptor | *fhuA* | 4 | 62.06 | 144.00 | 148.00 | IRON | up |
| 45 | XAC2936 | 27673 | 5.64 | 25598/5.71 | ABC transporter ATP-binding protein | *ynhD* | 5 | 1.04 | 4.60 | 4.37 | IRON | up |
| 62 | XAC3123 | 20391 | 5.71 | 21588/5.77 | DNA-bindingrelatedprotein | *dps* | 3 | 2.07 | 2.70 | 3.04 | IRON | up |
| 5 | XAC3201 | 81414 | 5.68 | 77150/5.63 | TonB-dependent receptor | *fyuA* | 3 | 2.57 | 1.56 | 0.48 | IRON | up |
| 128 | XAC3354 | 23033 | 6.1 | 21350/5.47 | Outermembraneprotein W | *ompW* | 4 | 0.00 | 0.00 | 0.00 | IRON | down |
| 89 | XAC3444 | 103243 | 4.79 | 89923/4.84 | TonB-dependent receptor | *btuB* | 10 | 1.78 | 0.00 | 0.00 | IRON | down |
| 93 | XAC3498 | 80719 | 5.70 | 72534/5.40 | Outer membrane receptor for ferric iron uptake | *fhuE* | 7 | 0.40 | 0.05 | 0.40 | IRON | down |
| 53 | XAC3664 | 23797 | 4.97 | 23610/4.83 | Outermembraneprotein | *ompW* | 4 | 2.28 | 4.08 | 3.36 | IRON | up |
| 87 | XAC4273 | 109344 | 5.21 | 89523/5.19 | OmpA-relatedprotein | *XAC4273* | 8 | 1.69 | 0.05 | 0.88 | IRON | down |
| 86 | XAC4274 | 110082 | 5.29 | 91340/5.21 | OmpA-relatedprotein | *XAC4274* | 9 | 1.66 | 0.27 | 0.43 | IRON | down |
| 3 | XAC4368 | 84447 | 5.51 | 80205/5.70 | TonB-dependent receptor | *fecA* | 4 | 0.00 | 8.99 | 3.16 | IRON | up |
| 16 | XAC0007 | 43415 | 6.79 | 37530/5.80 | Conserved hypothetical protein  (Putative Zn-dependent protease, contains TPR repeats) | *XAC0007* | 7 | 1.87 | 2.18 | 2.21 | ND | up |
| 71 | XAC0193 | 17603 | 5.35 | 18225/5.37 | Conserved hypothetical protein  (Phosphohistidine phosphatase SixA) | *XAC0193* | 3 | 14.45 | 10.25 | 2.40 | ND | up |
| 73 | XAC0381 | 15720 | 4.95 | 17469/5.00 | Conserved hypothetical protein  (Ketosteroid isomerase-related protein) | *XAC0381* | 3 | 1.69 | 3.55 | 0.80 | ND | up |
| 29 | XAC0470 | 34625 | 5.21 | 33252/5.29 | Phosphoribosylaminoimidazole-succinocarboxamidesynthase | *hemH* | 8 | 3.06 | 1.62 | 2.16 | ND | up |
| 110 | XAC0749 | 41321 | 5.46 | 42116/5.63 | GTP cyclohydrolase II/3,4-dihydroxy-2-butanone 4-phosphate synthase | *ribA* | 3 | 0.07 | 0.07 | 0.14 | ND | down |
| 27 | XAC1046 | 35739 | 5.4 | 34862/5.47 | Isocitratedehydrogenase | *icd* | 5 | 0.79 | 1.45 | 1.58 | ND | up |
| 57 | XAC1078 | 22839 | 5.39 | 22774/5.30 | ATP-dependent Clp protease proteolytic subunit | *clpP* | 6 | 2.71 | 1.10 | 3.01 | ND | up |
| 98 | XAC1321 | 53877 | 7.79 | 54431/6.17 | Periplasmic protease | *mucD* | 9 | 1.40 | 0.34 | 0.31 | ND | down |
| 19 | XAC1432 | 40558 | 5.53 | 38474/5.55 | Succinyl-diaminopimelatedesuccinylase | *dapE* | 8 | 0.92 | 1.68 | 8.40 | ND | up |
| 130 | XAC1643 | 19909 | 5.45 | 21350/5.65 | Poly(hydroxyalcanoate) granule associated protein | *phaF* | 7 | 0.00 | 0.00 | 0.00 | ND | down |
| 15 | XAC1650 | 45714 | 5.68 | 45222/5.81 | 3-phosphoshikimate 1-carboxyvinyltransferase | *aroA* | 3 | 24.03 | 0.00 | 3.49 | ND | up |
| 94 | XAC1716 | 61729 | 5.91 | 66074/5.95 | CTP synthetase | *pyrG* | 6 | 0.66 | 0.15 | 0.32 | ND | down |
| 12 | XAC1776 | 48508 | 5.31 | 45006/5.42 | Xyloseisomerase | *xylA* | 7 | 1.68 | 2.69 | 3.20 | ND | up |
| 14 | XAC1858 | 46126 | 6.16 | 41563/5.85 | Valine-pyruvateaminotransferase | *avtA* | 4 | 1.21 | 2.93 | 11.46 | ND | up |
| 79 | XAC1968 | 14132 | 5.03 | 17518/5.08 | Response regulator | *XAC1968* | 3 | 2.95 | 1.99 | 9.97 | ND | up |
| 119 | XAC2550 | 33347 | 4.96 | 35166/5.07 | Conserved hypothetical protein  (Proline racemase) | *XAC2550* | 7 | 0.00 | 0.00 | 0.00 | ND | down |
| 52 | XAC2736 | 23904 | 5.31 | 22987/5.37 | Carboxymethylenebutenolidase | *XAC2736* | 8 | 25.54 | 3.73 | 5.28 | ND | up |
| 69 | XAC3140 | 18214 | 5.95 | 19639/6.12 | Conserved hypothetical protein  (Periplasmic TolA-binding protein - function unknown) | *XAC3140* | 5 | 8.22 | 7.76 | 7.22 | ND | up |
| 47 | XAC3307 | 25269 | 5.61 | 26787/5.71 | Fumarylacetoacetatehydrolase | *XAC3307* | 4 | 3.52 | 1.52 | 18.46 | ND | up |
| 24 | XAC3344 | 36538 | 4.98 | 33667/5.16 | Fructose-bisphosphatealdolase | *XAC3344* | 5 | 5.99 | 2.48 | 2.97 | ND | up |
| 65 | XAC3437 | 19943 | 5.33 | 21870/5.27 | Adenylatekinase | *adk* | 7 | 1.21 | 1.66 | 1.82 | ND | up |
| 70 | XAC3652 | 18207 | 5.31 | 20005/5.35 | ATP synthase delta chain | *atpH* | 3 | 2.89 | 10.59 | 2.06 | ND | up |
| 63 | XAC3709 | 20070 | 6.4 | 20891/6.32 | Tryptophan repressor bindingprotein | *wrbA* | 3 | 5.06 | 4.43 | 0.00 | ND | up |
| 10 | XAC3851 | 49960 | 5.31 | 49587/5.38 | Conserved hypothetical protein  (Uncharacterized metalloenzymeYdcJ, glyoxalase superfamily) | *XAC3851* | 7 | 10.29 | 9.89 | 46.78 | ND | up |
| 39 | XAC3924 | 31875 | 4,94 | 32236/5.09 | Spermidinesynthase | *speE* | 6 | 7,57 | 1,07 | 1,99 | ND | up |
| 59 | XACa0018 | 22398 | 5.17 | 22887/5.28 | Partitionprotein A | *parA* | 6 | 2.69 | 1.33 | 2.62 | ND | up |
| 28 | XAC0288 | 34737 | 5.07 | 33229/5.25 | Oxidoreductase | *mocA* | 4 | 42.40 | 27.05 | 10.87 | REDOX | up |
| 26 | XAC0339 | 35920 | 5.54 | 35567/5.56 | Oxidoreductase | *XAC0339* | 7 | 4.23 | 17.89 | 10.42 | REDOX | up |
| 60 | XAC0554 | 21395 | 5.83 | 21753/5.97 | Nitroreductase | *XAC0554* | 9 | 22.22 | 0.56 | 0.53 | REDOX | up |
| 48 | XAC1160 | 24563 | 6.19 | 24931/6.19 | Oxidoreductase | *XAC1160* | 5 | 0.33 | 0.31 | 2.58 | REDOX | up |
| 6 | XAC2698 | 79970 | 6.24 | 80003/6.15 | NADH-ubiquinoneoxidoreductase, NQO3 subunit | *nuoG* | 6 | 5.21 | 13.89 | 1.78 | REDOX | up |
| 111 | XAC3802 | 41202 | 7.08 | 42046/6.22 | Conserved hypothetical protein  (Fe-S oxidoreductase, related to NifB/MoaA family) | *XAC3802* | 6 | 0.12 | 0.15 | 0.07 | REDOX | down |
| 20 | XAC1434 | 38703 | 5.89 | 35614/5.80 | Conserved hypothetical protein  (Nitrous oxidase accessory protein NosD, contains tandem CASH domains) | *XAC1434* | 5 | 13.37 | 54.21 | 13.10 | REDOX. | up |
| 25 | XAC4349 | 35994 | 5.95 | 35745/5.97 | Bifunctionaloxireductase/alginatelyase | *algL* | 3 | 2.79 | 0.12 | 3.43 | REDOX-A/B/LPS | up |
| 75 | XAC0282 | 14588 | 5.63 | 17375/5.63 | Organichydroperoxideresistanceprotein | *ohr* | 3 | 5.30 | 19.40 | 24.36 | ROS/OSM | up |
| 4 | XAC1301 | 82833 | 5.55 | 81968/5.65 | Catalase | *katG* | 8 | 11.52 | 19.27 | 35.31 | ROS/OSM | up |
| 31 | XAC2005 | 34530 | 5.71 | 29652/5.81 | Thioredoxinreductase | *trxB* | 2 | 1.90 | 0.99 | 2.29 | ROS/OSM | up |
| 64 | XAC2369 | 20069 | 6.08 | 19321/5.23 | General stress protein | *gsp* | 6 | 6.00 | 88.95 | 117.14 | ROS/OSM | up |
| 58 | XAC2386 | 22703 | 5.47 | 23236/2.47 | Superoxidasedismutase | *sodM* | 3 | 1.45 | 1.84 | 1.75 | ROS/OSM | up |
| 38 | XAC2783 | 31423 | 4.61 | 29856/4.81 | Thioredoxin | *trx* | 6 | 2.93 | 1.89 | 2.42 | ROS/OSM | up |
| 74 | XAC2915 | 15400 | 5.59 | 17556/5.60 | Osmoticallyinducibleprotein | *osmC* | 4 | 0.95 | 2.89 | 2.89 | ROS/OSM | up |
| 32 | XAC3103 | 34528 | 5.76 | 33950/5.68 | Glutathionesynthetase | *gshB* | 8 | 1.09 | 0.61 | 2.75 | ROS/OSM | up |
| 66 | XAC4346 | 19910 | 5.16 | 22910/5.28 | Glutathioneperoxidase | *btuE* | 2 | 24.25 | 1.73 | 13.30 | ROS/OSM | up |
| 23 | XAC0656 | 37601 | 5.64 | 38915/5.69 | Rod shape-determiningprotein | *mreB* | 5 | 0.96 | 3.08 | 8.57 | VIR-ADAP | up |
| 44 | XAC0834 | 28176 | 6.03 | 25267/5.67 | Two-component system regulatory protein | *colR* | 8 | 1.58 | 2.01 | 2.50 | VIR-ADAP | up |
| 54 | XAC1028 | 23680 | 5.98 | 25363/6.10 | Phosphoglyceratemutase | *pgmA* | 3 | 3.46 | 6.88 | 0.77 | VIR-ADAP | up |
| 67 | XAC2932 | 19457 | 4.73 | 22108/4.89 | Protease | *pfpI* | 6 | 6.09 | 11.82 | 14.43 | VIR-ADAP | up |
| 9 | XAC3300 | 62268 | 4.94 | 57442/5.40 | Lipase | *estA* | 4 | 1.93 | 21.98 | 96.64 | VIR-ADAP | up |
| 21 | XAC3456 | 38289 | 5.27 | 38194/5.33 | 3-isopropylmalatedehydrogenase | *leuB* | 5 | 43.75 | 7.62 | 18.57 | VIR-ADAP | up |
| 49 | XAC3457 | 24408 | 5.23 | 24082/5.30 | 3-isopropylmalatedehydratasesmallsubunit | *leuD* | 5 | 65.18 | 2.46 | 8.27 | VIR-ADAP | up |
| 81 | XAC3671 | 18304 | 5.97 | 19879/ 6.16 | Conserved hypothetical protein  (Uncharacterized conserved protein YajQ, UPF0234 family) | *XAC3671* | 5 | 8.74 | 84.52 | 0.19 | VIR-ADAP | up |
| 35 | XAC4009 | 33388 | 5.12 | 33574/5.23 | Arginase | *argI* | 6 | 1.34 | 8.41 | 11.58 | VIR-ADAP | up |
| 30 | XAC4109 | 34589 | 5.81 | 29652/5.81 | Aerobiccoproporphyrinogen III oxidase | *hemF* | 3 | 3.23 | 0.24 | 1.70 | VIR-ADAP | up |
| 13 | XACb0007 | 46215 | 5.89 | 44072/5.44 | Lyticmureintransglycosylase | *mlt/MltB* | 7 | 8.11 | 71.06 | 113.00 | VIR-ADAP | up |
