# Supplementary material for: Proteomics-based identification of differentially abundant proteins reveals adaptation mechanisms of Xanthomonas citri subsp. citri during Citrus sinensis infection
Source: BMC Microbiol. 2017 Jul 11;17:155. doi: 10.1186/s12866-017-1063-x (PMC5504864; doi:10.1186/s12866-017-1063-x)
Supplement: Supplementary file 3 — Profile of TonB receptors Up and Down regulated in infectious conditions. (DOCX 17 kb) [file 12866_2017_1063_MOESM3_ESM.docx]

Supplementary Material 3

**Proteomic analysis reveals adaptation mechanisms of *Xanthomonascitri* subsp. *citri* during initial stages of *Citrus sinensis* infection**

Leandro M Moreira^1,2^, Márcia R Soares^3^, Agda P Facincani^4^, Cristiano B Ferreira^4^, Rafael M Ferreira^4^, Maria I T Ferro^4^, Fábio C Gozzo^5^, Érica B Felestrino^2^, RenataA B Assis^2^, Camila Carrião Machado Garcia^1,2^, João C Setubal^6,8^, Jesus A. Ferro^4^, Julio C.F. de Oliveira^7^

**Supplemental Table2:** Profile of TonB receptors Up and Down regulated in infectious conditions.

| **Gene Id** | **Gene**  **Name** | **Product**  **(annotation)** | **Target** | **H.CUT^a^** | **Expres.** |
| --- | --- | --- | --- | --- | --- |
| **XAC0176** | *fpvA* | Ferripyoverdine receptor | Ferripioverdin | Furregulated TBDR (XCC0158) | Down |
| **XAC0823** | *phuR* | Outermembranehemin receptor | Hemin | Furregulated TBDR (XCC0768) | Down |
| **XAC2742** | *btuB* | TonB-dependent receptor | Vitamin B12 | XCC2572 | Down |
| **XAC2743** | *oar* | Oarprotein | ---- | Ps-TBDR/oar XCC2573 | Down |
| **XAC2829** | *phuR* | Outer Membrane hemin receptor | Hemim | XCC2658 | Up |
| **XAC2830** | *fhuA* | TonB dependent receptor | Ferrichrome | XAC2665 | Up |
| **XAC3201** | *fyuA* | TonB dependent receptor | Fe3+Yersiniabactin | ---- | Up |
| **XAC3354** | *ompW* | Outermembraneprotein W | ---- | ---- | Down |
| **XAC3444** | *btuB* | TonB-dependent receptor | Vitamin B12 | XCC3316 | Down |
| **XAC3498** | *fhuE* | Ferric iron uptake outer membrane protein | Ferric Iron | ---- | Down |
| **XAC3664** | *ompW* | Outermembraneprotein W | ---- | ---- | Up |
| **XAC4273** | *ompA* | OmpA-related protein | ---- | Putative partial CUT locus (XCC4131)  Ps-TBDR/oar | Down |
| **XAC4274** | *ompA* | OmpA-related protein | ---- | Putative partial CUT locus (XCC4132)  Ps-TBDR/oar | Down |
| **XAC4368** | *fecA* | TonB dependent receptor | Ferric citrate | Putative partial CUT locus, CAZy associated TBDR, xylan induced TBDR (XACC4235) | Up |

a Homologous CUT regions based on Xcc genome analysis [[65](#_ENREF_65)].
