# Supplementary material for: Proteomics-based identification of differentially abundant proteins reveals adaptation mechanisms of Xanthomonas citri subsp. citri during Citrus sinensis infection
Source: BMC Microbiol. 2017 Jul 11;17:155. doi: 10.1186/s12866-017-1063-x (PMC5504864; doi:10.1186/s12866-017-1063-x)
Supplement: Supplementary file 4 — Energy metabolism of Xac highlighting a set of proteins down-regulated in infectious conditions. (DOCX 783 kb) [file 12866_2017_1063_MOESM4_ESM.docx]

Supplementary Material 4

**Proteomics-based identification of differentially abundant proteins reveals adaptation mechanisms of *Xanthomonas citri* subsp. *citri* during *Citrus sinensis* infection.**

Leandro M Moreira^1,2^, Márcia R Soares^3^, Agda P Facincani^4^, Cristiano B Ferreira^4^, Rafael M Ferreira^4^, Maria I T Ferro^4^, Fábio C Gozzo^5^, Érica B Felestrino^2^, RenataA B Assis^2^, Camila Carrião Machado Garcia^1,2^, João C Setubal^6,8^, Jesus A. Ferro^4^, Julio C.F. de Oliveira^7^

**
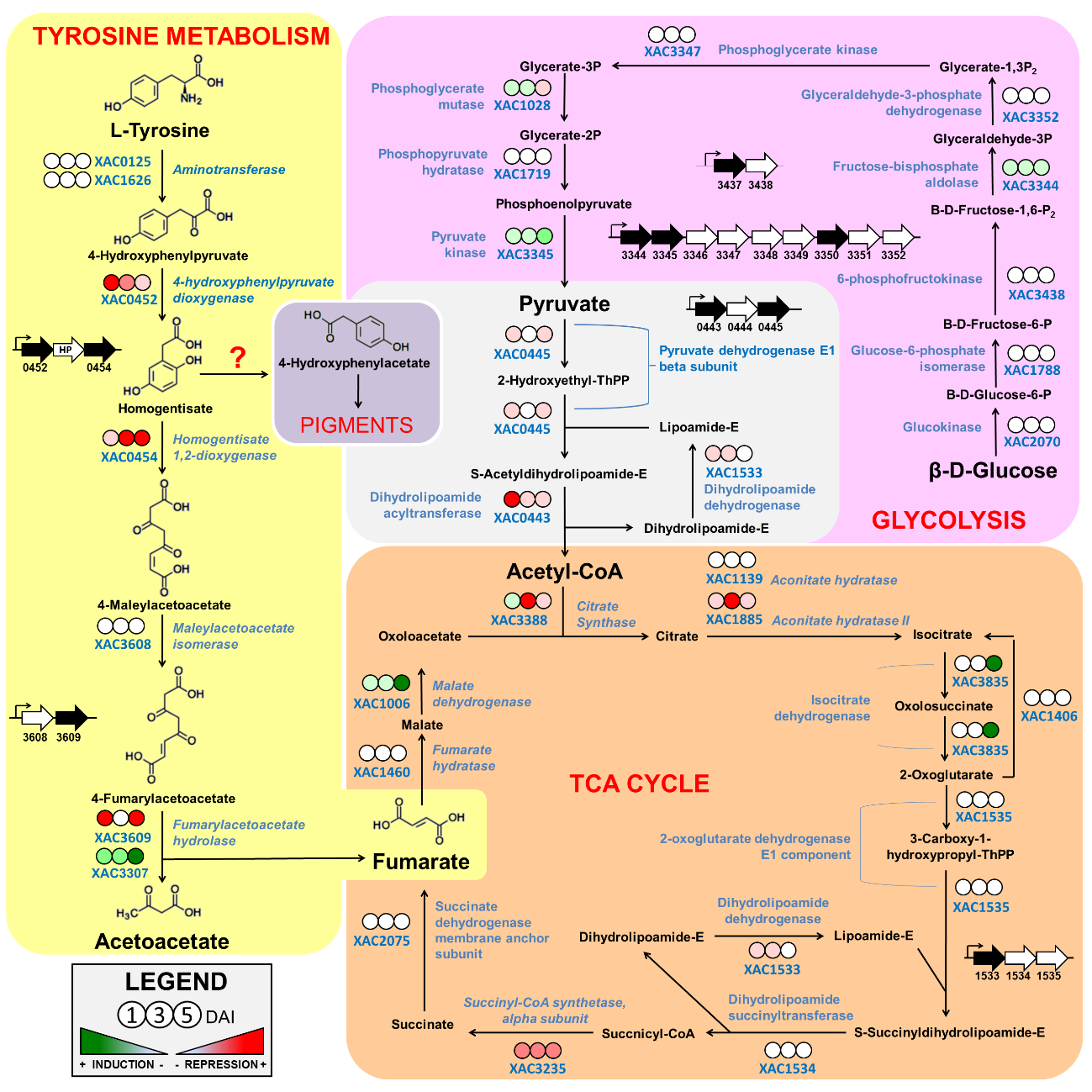
**

**Supplemental Figure 2. Energy metabolism of Xac highlighting a set of proteins down-regulated in infectious conditions.** Three out of five related tyrosine metabolism proteins (yellow background) were negatively regulated, so does all the pyruvate dehydrogenase complex (blue background) and three enzymes involved in the tricarboxylic acid cycle (orange background). The black arrows highlight the genes encoding the respective proteins down-regulated in infectious conditions, some of which apparently all composing a repressed operon.
