# Supplementary material for: Proteomics-based identification of differentially abundant proteins reveals adaptation mechanisms of Xanthomonas citri subsp. citri during Citrus sinensis infection
Source: BMC Microbiol. 2017 Jul 11;17:155. doi: 10.1186/s12866-017-1063-x (PMC5504864; doi:10.1186/s12866-017-1063-x)
Supplement: Supplementary file 5 — Expression profile of Hypothetical and Conserved hypothetical proteins in infectious conditions. (DOCX 15 kb) [file 12866_2017_1063_MOESM5_ESM.docx]

Supplementary Material 5

**Proteomic analysis reveals adaptation mechanisms of *Xanthomonascitri* subsp. *citri* during initial stages of *Citrus sinensis* infection**

Leandro M Moreira^1,2^, Márcia R Soares^3^, Agda P Facincani^4^, Cristiano B Ferreira^4^, Rafael M Ferreira^4^, Maria I T Ferro^4^, Fábio C Gozzo^5^, Érica B Felestrino^2^, RenataA B Assis^2^, Camila Carrião Machado Garcia^1,2^, João C Setubal^6,8^, Jesus A. Ferro^4^, Julio C.F. de Oliveira^7^

**Supplemental Table 3:** Expression profile of Hypothetical and Conserved hypothetical proteins in infectious conditions.

| **SPOT** | **PROTEIN** | **PROD ANNOTATION** | **COG** | **COG Prod** | **Expression** | **XAM1/NB** | **3DAI/NB** | **5DAI/NB** | **CLASS** |
| --- | --- | --- | --- | --- | --- | --- | --- | --- | --- |
| 76 | XAC1093 | CHP | No COG | ---- | Up | 2.85 | 1.56 | 6.47 | HYP |
| 72 | XAC1364 | CHP | No COG | ---- | Up | 2.93 | 3.48 | 5.45 | HYP |
| 55 | XAC2246 | HP | No COG | ---- | Up | 5.18 | 4.42 | 4.72 | HYP |
| 84 | XAC3680 | CHP | No COG | ---- | Up | 1.69 | 3.55 | 0.80 | HYP |
| 80 | XAC3866 | CHP | No COG | ---- | Up | 5.33 | 54.29 | 76.17 | HYP |
| 82 | XAC3981∆ | CHP | No COG | ---- | Up | 21.26 | 21.43 | 21.36 | HYP |
| 83 | XAC2315# | CHP | 2350 | Uncharacterized conserved protein YciI, contains a putative active-site phosphohistidine | Up | 8.79 | 2.48 | 1.92 | HYP |

* Reannotated, # present putative functional domains previously described, ∆ mutant analyzed.
