# Supplementary material for: Proteomics-based identification of differentially abundant proteins reveals adaptation mechanisms of Xanthomonas citri subsp. citri during Citrus sinensis infection
Source: BMC Microbiol. 2017 Jul 11;17:155. doi: 10.1186/s12866-017-1063-x (PMC5504864; doi:10.1186/s12866-017-1063-x)
Supplement: Supplementary file 6 — Phylogenetic and String analysis of Xanthomonas conserved hypothetical protein related to new possible pathogenicity island. (DOCX 615 kb) [file 12866_2017_1063_MOESM6_ESM.docx]

Supplementary Material 6

**Proteomics-based identification of differentially abundant proteins reveals adaptation mechanisms of *Xanthomonas citri* subsp. *citri* during *Citrus sinensis* infection.**

Leandro M Moreira^1,2^, Márcia R Soares^3^, Agda P Facincani^4^, Cristiano B Ferreira^4^, Rafael M Ferreira^4^, Maria I T Ferro^4^, Fábio C Gozzo^5^, Érica B Felestrino^2^, RenataA B Assis^2^, Camila Carrião Machado Garcia^1,2^, João C Setubal^6,8^, Jesus A. Ferro^4^, Julio C.F. de Oliveira^7^


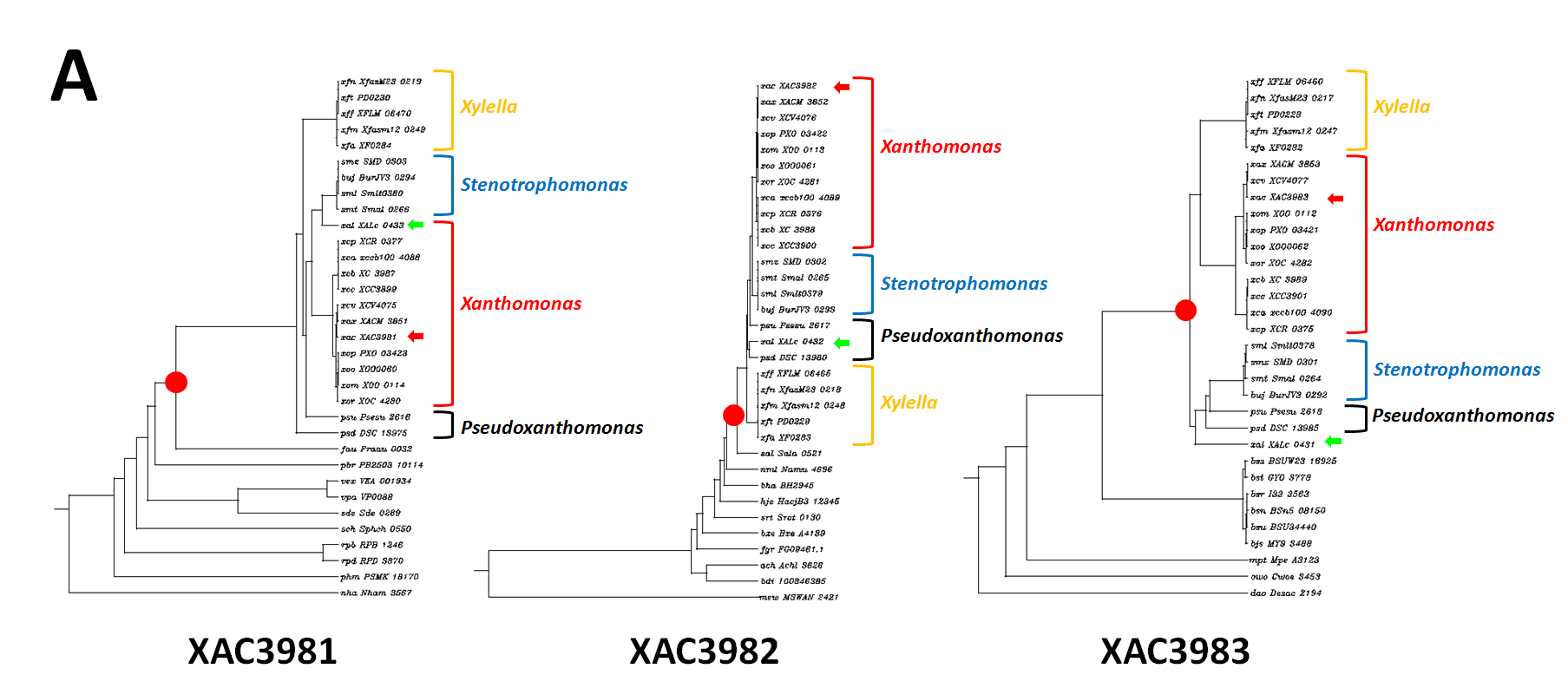


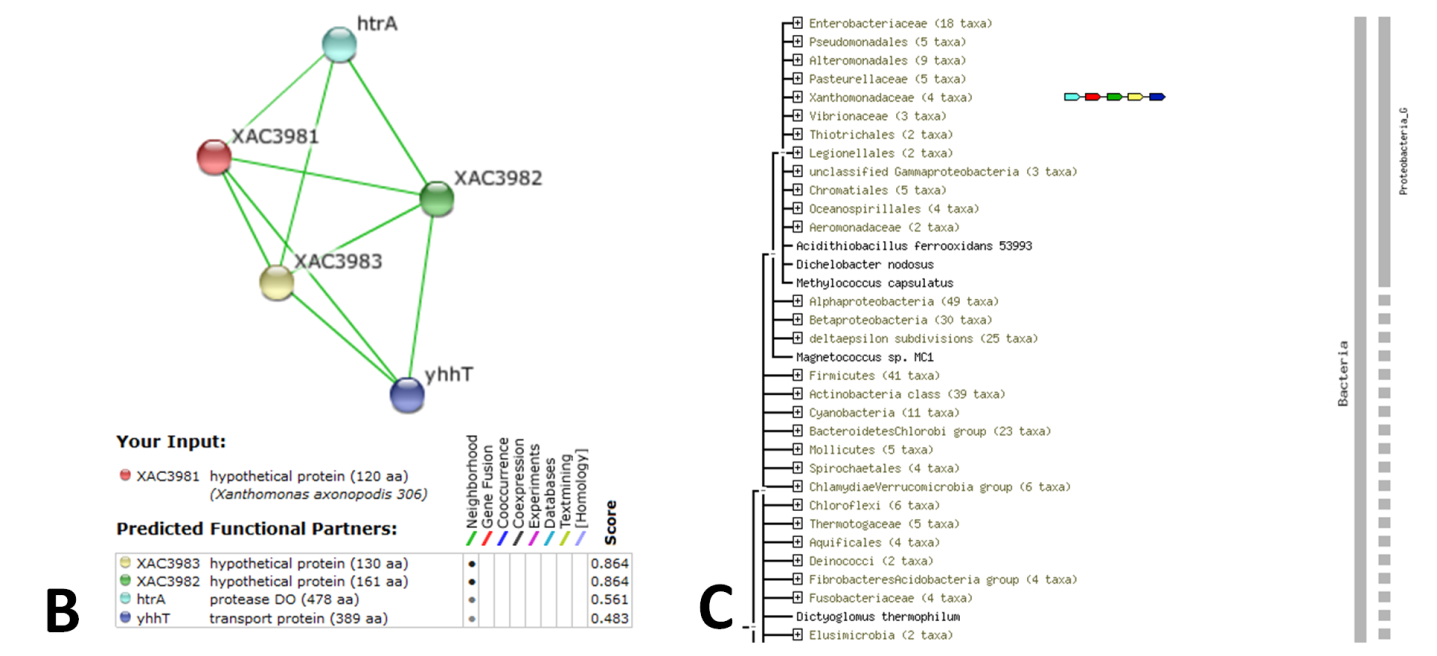


**A.Phylogenetic analysis of *Xanthomonas* conserved hypothetical proteins related to the operon XAC3980-3983.** The colors highlight the different genera belonging to Xanthomonadaceae family. The red circle highlights the separation between Xanthomonadaceaefamily (above) and other organisms used as reference (below). The red arrow highlights the respective genes from Xac while the green arrow highlights the respective genes of *X. albilineans* (Xalb). Note that in Xalb each gene occupies a different position in the respective clades.

**B – C.String analysis of *Xanthomonas* conserved hypothetical proteins related to a new possible pathogenicity island.** The interaction network highlights only the genes that are in the neighborhood of the reference gene (XAC3981), without any other relationship described in the literature between these genes (B). This gene syntenyis observed only in organisms from the genus *Xanthomonas*(C).
